# Wnt/β-catenin signaling regulates VE-cadherin-mediated anastomosis of brain capillaries by counteracting S1pr1 signaling

**DOI:** 10.1038/s41467-018-07302-x

**Published:** 2018-11-19

**Authors:** Kathleen Hübner, Pauline Cabochette, Rodrigo Diéguez-Hurtado, Cora Wiesner, Yuki Wakayama, Kathrin S. Grassme, Marvin Hubert, Stefan Guenther, Heinz-Georg Belting, Markus Affolter, Ralf H. Adams, Benoit Vanhollebeke, Wiebke Herzog

**Affiliations:** 1 University of Muenster, Schlossplatz 2, 48149 Muenster, Germany; 2 Cells-in-Motion Cluster of Excellence (EXC 1003 – CiM), University of Muenster, Waldeyerstrasse 15, 48149 Muenster, Germany; 3 Université libre de Bruxelles, Rue Prof. Jeener et Brachet 12, 6041 Gosselies, Belgium; 4 Max Planck Institute for Molecular Biomedicine, Roentgenstrasse 20, 48149 Muenster, Germany; 5 Biozentrum der Universität Basel, Klingelbergstrasse 70, 4056 Basel, Switzerland; 6 Max Planck Institute for Heart and Lung Research, ECCPS Bioinformatics and Deep Sequencing Platform, Ludwigstrasse 43, 61231 Bad Nauheim, Germany; 7 Walloon Excellence in Life Sciences and Biotechnology (WELBIO), Avenue Pasteur 6, 1300 Wavre, Belgium

## Abstract

Canonical Wnt signaling is crucial for vascularization of the central nervous system and blood-brain barrier (BBB) formation. BBB formation and modulation are not only important for development, but also relevant for vascular and neurodegenerative diseases. However, there is little understanding of how Wnt signaling contributes to brain angiogenesis and BBB formation. Here we show, using high resolution in vivo imaging and temporal and spatial manipulation of Wnt signaling, different requirements for Wnt signaling during brain angiogenesis and BBB formation. In the absence of Wnt signaling, premature Sphingosine-1-phosphate receptor (S1pr) signaling reduces VE-cadherin and Esama at cell-cell junctions. We suggest that Wnt signaling suppresses S1pr signaling during angiogenesis to enable the dynamic junction formation during anastomosis, whereas later S1pr signaling regulates BBB maturation and VE-cadherin stabilization. Our data provides a link between brain angiogenesis and BBB formation and identifies Wnt signaling as coordinator of the timing and as regulator of anastomosis.

## Abstract (web summary)

Wnt signaling is known to regulate the formation of the blood-brain barrier. Here Hübner et al. dissect the underlying mechanisms using high resolution live imaging in zebrafish, and find that Wnt regulates anastomosis of angiogenic sprouts in the brain by counteracting sphingosine-1-phosphate receptor signaling.

## Introduction

**Fig. 1:**
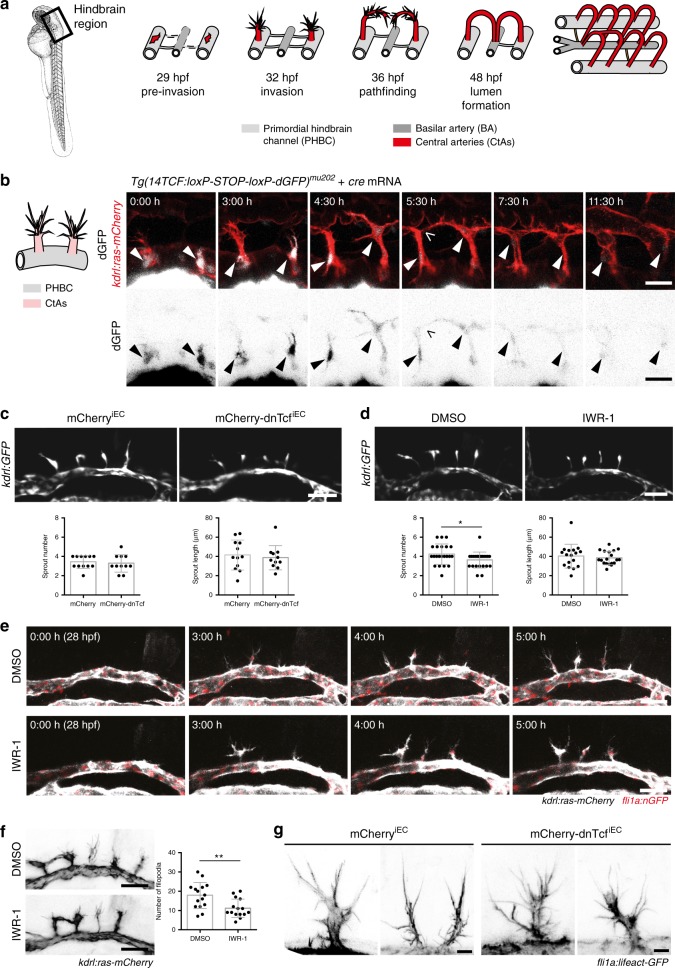
Wnt signaling is not required for migration of brain capillary sprout ECs. **a** Illustration of hindbrain angiogenesis in zebrafish embryos. Black box indicates localization of the hindbrain. Brain capillary (central artery, CtA) pre-tip cells can be detected within two bilateral primordial hindbrain channels (PHBCs, light gray) and sprout dorsally from PHBCs around 32 hpf. Between 32 and 36 hpf, CtA sprouts extend long filopodia and migrate in an arch toward the basilar artery (BA, dark gray). At 48 hpf CtAs have fused with either the BA or neighboring CtAs and carry blood flow. **b** Time-lapse analysis of a *cre* mRNA-injected *Tg(14TCF:loxP-STOP-loxP-dGFP)*^*mu202*^ embryo starting from around 29 hpf showed continuously active Wnt signaling in CtAs (arrowheads) before and during sprouting, during invasion, cell–cell contact (open arrowhead) and lumen formation. The bottom panel represents single channel images in inverted color for better visualization. **c**, **d** Inhibition of Wnt signaling by EC-specific dnTcf expression after heat shock at 26 hpf (mCherry-dnTcf^iEC^, **c**) or pharmacologically by IWR-1 treatment (**d**) resulted in normal CtA sprouting at 32 hpf. CtA sprout number or length of the sprouts was not affected by mCherry-dnTcf^iEC^ expression in *Tg(kdrl:GFP)*^*s843*^ embryos (**c**: mCherry-dnTcf^iEC^: *n* = 11; mCherry^iEC^: *n* = 12) or IWR-1 treatment (**d**: DMSO: number *n* = 22, length *n* = 17; IWR-1: number *n* = 24, length *n* = 18). **e** Still images from time-lapse movies starting at around 28 hpf displayed normal CtA sprout formation in *Tg(kdrl:ras-mCherry)*^*s896*^*; (fli1a:nGFP)*^*y7*^ double transgenic embryos treated with IWR-1. **f**, **g** CtA sprout morphology was not affected by IWR-1 treatment (**f**) or dnTcf expression (**g**) in *Tg(fli1a:lifeact-GFP)*^*mu240*^ embryos. IWR-1 treatment slightly reduced the formation of long filopodia (>10 µm) compared to DMSO control (**f**: IWR-1: *n* = 15; DMSO: *n* = 15). Images are displayed in inverted color for better visualization. Confocal images show dorsal (**b**) or lateral views (**c**–**g**), anterior to the left. Values represent mean ± SD. *p < 0.05, **p < 0.01, ***p < 0.001, Student’s *t*-test; *n*, number of analyzed embryos; BA, basilar artery; CtAs, central arteries; ECs, endothelial cells; hpf, hours post fertilization; PHBC, primordial hindbrain channel; Scale bars: 30 µm (**b**), 50 µm (**c**–**f**), 6 µm (**g**)

The central nervous system (CNS) depends on nutrient and oxygen delivery from blood vessels during the development and homeostasis, but also requires protection from blood-born toxins and pathogens. Endothelial cells (ECs) of CNS blood vessels acquire characteristic properties in order to fulfill the tasks of this blood-brain barrier (BBB), such as expression of a specific subset of junction molecules and nutrient transporters, downregulation of vesicular transport and establishment of cell–cell interactions within the neurovascular unit^1^. During a defined time window of embryonic development, molecular cues from neuronal and perineuronal tissues orchestrate CNS angiogenesis and barriergenesis^2–4^. The process of brain angiogenesis is well conserved in vertebrates: After acquiring a pre-sprouting signature (hereafter called pre-tip cell), these specified cells migrate out from the resident vessel, with tip cells guiding sprout formation, and invade into the neuronal tissue, where they form cell–cell contacts and anastomose with other sprouts or extra-cerebral vessels in order to establish circulatory loops^1,3,5^. In zebrafish (*Danio rerio*), hindbrain capillaries (intra-cerebral central arteries, CtAs) invade the brain parenchyma at around 32 h post fertilization (hpf) from the primordial hindbrain channel (PHBC), extend dorsally and connect ventrally to the basilar artery (BA) or laterally to other CtAs (Fig. 1a)^4–7^. At 48 hpf, most CtAs carry blood flow and by 72 hpf most of the respective BBB properties are established^8,9^.

Studies in mice and zebrafish demonstrate β-catenin-dependent Wnt signaling through Wnt7a–Wnt7b to be essential for brain vascularization^10–12^. Additionally, Wnt signaling has been shown to be involved in the establishment of BBB characteristics, such as upregulation of tight junction components (e.g., Claudin 1 and 3) or nutrient transporters (e.g., Glut1) and decrease of transcellular transport processes (e.g., Plvap)^10,13–15^. Recently, the Gpr124-Reck complex has been identified as an EC autonomous regulator of Wnt signaling essential for brain vascularization and BBB formation^12,15–20^. Single-cell analysis of brain vascular development through live imaging approaches in zebrafish further revealed that the control of pre-tip cell function by the Gpr124-Reck complex operates within the parental vessels (PHBCs) at pre-invasive stages. This early function of Wnt signaling has so far largely precluded the analysis of Wnt signaling functions at later stages of CNS vascular development, including the invasion process itself and subsequent patterning events.

Vascular endothelial (VE)-cadherin is the major endothelial-specific member of the cadherin protein family and is involved in EC migration, cell–cell contact formation, anastomosis, and barrier formation^5,21,22^. Together with the EC-selective adhesion molecule a (Esama), VE-cadherin is essential for EC–EC recognition and contact formation during intersegmental vessel anastomosis^23,24^. Experiments using mice and tissue culture revealed that VE-cadherin is subject to extensive post-transcriptional regulation, including intracellular complex formation and trafficking, membrane localization as well as association in cell–cell junctions. This dynamic regulation of VE-cadherin in adherens junctions is not only crucial for vascular patterning, but also for the function of mature blood vessels, e.g., for maintenance of EC integrity or during leukocyte extravasation^25,26^, and can be regulated by Sphingosine-1-phosphate (S1p) signaling^27,28^.

ECs express S1p receptor (S1pr) 1, 2, and 3^29^ and in zebrafish, two paralogues exist for S1pr3 (S1pr3a and S1pr3b). S1pr1 signaling promotes BBB integrity in mice^30^. Additionally, postnatal EC-specific knockout of S1pr1 induces loss of VE-cadherin and vascular endothelial growth factor receptor 2 (VEGFR2) from the cell junctions^31^. To date, the contribution of neither S1pr nor VE-cadherin signaling has been addressed during brain capillary angiogenesis, leaving a missing link between brain EC angiogenesis and BBB formation.

In this study, we clarify the distinct requirements for Wnt signaling during brain angiogenesis. Whereas Wnt signaling is essential before sprouting to regulate yet to be defined early function of the future tip cells (pre-tip cells) within the parental vessel^12^, it is surprisingly dispensable during sprout elongation and migration, although it remains continuously active in the invading sprout. We show that Wnt signaling is regulating brain capillary anastomosis and lumen formation. Interestingly, Wnt signaling is crucial for VE-cadherin and Esama localization at cell–cell junctions in a transcription-independent manner. During active brain capillary angiogenesis, Wnt signaling counteracts S1pr signaling, which enables VE-cadherin-dependent anastomosis and lumen formation. In contrast, when angiogenesis is completed at later stages, S1pr signaling regulates BBB formation. Our data therefore reveal an important functional link between the intertwined processes of brain capillary angiogenesis and BBB formation.

## Results

### Wnt signaling is required for hindbrain capillary patterning

Studies in mice have shown that Wnt signaling is essential for hindbrain capillary angiogenesis^10,11,14^. Studies using zebrafish revealed that this control by Wnt signaling reflects an essential function in defining the pre-tip cell during early, pre-invasive angiogenic events within the parental vessels (Fig. 1a^12^). As a result, no hindbrain capillaries (central arteries, CtA) are formed in the absence of Wnt signaling. In order to bypass this early requirement, we blocked Wnt signaling pharmacologically using IWR-1^32^ and generated transgenic lines, which allow for temporally and spatially controlled expression of a dominant-negative Tcf transcription factor.

Treatment with Wnt signaling inhibitor IWR-1 after the pre-tip cell specification phase (29–72 hpf) resulted in severe vascular defects and hemorrhages in the brain, but not in the trunk vasculature (Supplementary Fig. 1a), indicating that there are further requirements for Wnt signaling during brain angiogenesis. We therefore analyzed during which stages of brain capillary angiogenesis active Wnt signaling can be observed using confocal live imaging and two different transgenic Wnt signaling reporter lines, which express short-lived fluorophores controlled by Wnt-responsive promoters: *Tg(axin2BAC:Venus-Pest)*^*mu288*^ and *Tg(14TCF:loxP-STOP-loxP-dGFP)*^*mu202*^^33^. Both reporter lines showed robust fluorophore expression within the migrating CtA sprouts at 34 hpf (Supplementary Fig. 1b,c), which we confirmed to be EC specific (Supplementary Fig. 1d). We next performed time-lapse analysis using *cre* mRNA-injected *Tg(14TCF:loxP-STOP-loxP-dGFP)*^*mu202*^ embryos starting from 29 hpf (Fig. 1b, Supplementary movies 1, 2). We detected high Wnt reporter activity in the determined CtA pre-tip cells, which are forming within the PHBC. Despite a putative half-life of about 2 h, the destabilized GFP (dGFP) expression remained high in the emerging CtA sprouts during the following 5.5 h of development, indicating continuous Wnt signaling activity during CtA sprout migration and cell–cell contact formation (open arrow, Fig. 1b). With the onset of lumen formation about 2 h after cell–cell-contact formation, the dGFP signal decreased to a baseline level, which was maintained in the perfused CtAs, pointing to a post-determination role of Wnt signaling.

After pre-tip cell formation, CtA angiogenesis proceeds by sprouting and sprout invasion into the brain parenchyma. We asked whether endothelial Wnt signaling regulates these processes.

We therefore generated a transgenic zebrafish line expressing a dominant-negative Tcf transcription factor fused to mCherry (mCherry-dnTcf) under the control of a heat shock inducible promoter (hsp70l). To restrict Wnt inhibition to ECs, we placed a loxP-flanked (floxed) STOP-cassette upstream of the mCherry-dnTcf coding sequence (Supplementary Fig. 2a, c). This STOP cassette was removed only in ECs by mating to fish with endothelial-specific Cre recombinase expression (*Tg(kdrl:cre)*^*s898*^). Validation of Wnt inhibition by heat shock controlled mCherry-dnTcf expression is documented in Supplementary Fig. 2.

To address whether Wnt signaling regulates sprout elongation or EC migration, we blocked Wnt signaling by EC-specific mCherry-dnTcf expression or IWR-1 treatment starting from 26 hpf and analyzed CtA sprouts at 32 hpf (Fig. 1c, d). Surprisingly, we did not observe differences in CtA sprout number or sprout length after Wnt signaling inhibition (Fig. 1c, d). Furthermore, sprout formation, behavior, and filopodia appearance were not affected by IWR-1 treatment (Fig. 1e, f, Supplementary movies 3, 4). Only the formation of long filopodia (>10 µm) was marginally decreased after IWR-1 treatment (Fig. 1f). Similarly, heat shock-induced EC-specific overexpression of mCherry-dnTcf (mCherry-dnTcf^iEC^) did not result in differences in filopodia morphology, but instead in high filopodia motility, which resulted in slightly blurry images due to fast filopodia movement (Fig. 1g).

We conclude that after being required early within the parental vessel, Wnt signaling is continuously active during CtA angiogenesis, but dispensable for efficient sprout elongation and invasion of CtA tip cells into the brain parenchyma.

### Wnt regulates brain capillary anastomosis and lumenization

**Fig. 2:**
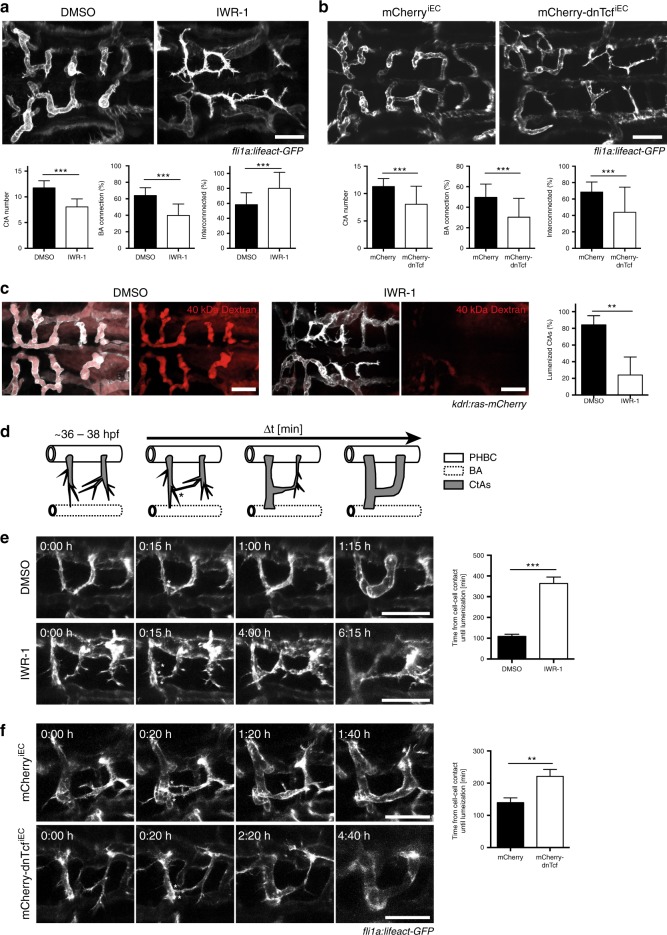
Wnt signaling is crucial for brain capillary patterning and anastomosis. **a**, **b** Inhibition of Wnt signaling by IWR-1 treatment (**a**) or dnTcf expression (**b**) impaired CtA patterning. **a** IWR-1 treatment from 29 to 48 hpf reduced CtA number (*n* = 39) and the proportion of CtAs connecting to the BA (*n* = 39), but increased the proportion of interconnecting CtAs (*n* = 33), compared to DMSO treated embryos (CtA number: *n* = 35; BA connection: *n* = 35; interconnected: *n* = 29). **b** Expression of mCherry-dnTcf^iEC^ through heat shock at 26 hpf reduced the number of CtAs (*n* = 40) and decreased the proportion of CtAs connecting to the BA (*n* = 32) or interconnecting (*n* = 32), compared to mCherry^iEC^ control at 48 hpf (CtA number: *n* = 39; BA connection: *n* = 33; interconnected: *n* = 33). **c** Analysis of lumen formation by fluorescent tracer injection into the blood stream at 48 hpf. Treatment with IWR-1 decreased the proportion of lumenized CtAs (*n* = 16) compared to DMSO control (*n* = 18). **d** Schematic representation of CtA anastomosis (dorsal view, ~36 hpf). CtA sprouts (gray) migrate, extend filopodia and form cell–cell contacts to neighboring CtA sprouts (asterisk) or the BA. Cell–cell contact formation triggers anastomosis, cell rearrangements and lumen formation through the connected sprouts in a distinct time window (Δt). **e**, **f** Inhibition of Wnt signaling drastically extended the time window from cell–cell contact formation until lumen formation. Still images from time-lapse movies embryos treated with DMSO (**e**: *n* = 58, *N* = 9) or IWR-1 (**e**: *n* = 33, *N* = 9) or expressing mCherry^iEC^ (**f**: *n* = 36, *N* = 6) or mCherry-dnTcf^iEC^ (**f**: *n* = 39, *N* = 8) after heat shock treatment at 26 hpf. Note that for events, where lumen formation was not completed until the end of the time-lapse recording (~48 hpf), the last measured time point was used for quantification. Confocal images show dorsal views (anterior to the left) of *Tg(fli1a:lifeact-GFP)*^*mu240*^ (**a**, **b**, **e**, **f**) or *Tg(kdrl:ras-mCherry)*^*s896*^ (**c**) embryos. Values represent mean ± SD (**a**–**c**) or ± SEM (**e**, **f**). *p < 0.05, **p < 0.01, ***p < 0.001, Student’s *t*-test; *n* number of analyzed embryos (**a**–**c**) or *n* number of CtA fusion events analyzed; *N*, number of embryos analyzed (**e**, **f**). BA, basilar artery; CtAs, central arteries; ECs, endothelial cells; Scale bars: 50 µm

To address the role of the active Wnt signaling in the developing CtAs, we inhibited Wnt signaling by inducing mCherry-dnTcf expression in ECs at 26 hpf or by applying IWR at 29 hpf and analyzed CtA pattern and lumen formation at 48 hpf (Fig. 2a, b). We observed a reduced CtA number, reduced connections to the BA and ectopic connections between CtAs. CtA patterning appeared more disorganized, and CtAs had non-lumenized protrusions. To address whether the reduced number of CtAs (Fig. 2a, b) correlated to a reduced number of EC within CtAs, we blocked Wnt signaling as before, and quantified the number of cell nuclei in *Tg(fli1a:nGFP)*^*y7*^ embryos. Both, treatment with IWR-1 or heat shock-induced EC-specific mCherry-dnTcf expression, reduced the EC number within CtAs (Supplementary Fig. 3a, b). However, blocking cell proliferation via the administration of aphidicolin and hydroxyurea (AHU) did not result in any patterning defects in CtAs, and the combined inhibition of Wnt signaling and proliferation did not aggravate the phenotype of Wnt deficiency (Supplementary Fig. 3c). We therefore conclude that Wnt signaling is required for CtA patterning, but does not act via cell proliferation.

To understand the processes underlying the CtA patterning defects observed after Wnt signaling inhibition (Fig. 2a, b), we performed angiography following IWR-1 treatment (Fig. 2c). In control embryos, more than 80% of the CtAs were perfused at 48 hpf, whereas after IWR-1 treatment only about 25% of the CtAs carried the fluorescent tracer. We next performed time-lapse analysis following IWR-1 treatment or EC-specific mCherry-dnTcf expression using *Tg(fli1a:lifeact-GFP)*^*mu240*^ embryos to illustrate the actin cytoskeleton of ECs (Fig. 2d–f). We focused on laterally connecting CtAs for better visualization and quantified the time from cell–cell contact until completion of lumen formation until 48 hpf (Fig. 2d). In control embryos, the average time from cell–cell contact to lumen formation is less than 2 h (Fig. 2d, e). After inhibition of Wnt signaling by IWR-1, we noted a drastic delay in lumen formation or even completely impaired lumenization within the observation window (Fig. 2e; Supplementary movie 5-8). Additionally, tip cells in CtA sprouts of IWR-1-treated embryos failed to establish stable cell–cell contacts, but instead continuously produced filopodia, leading to the formation of multiple cell–cell contact sites (asterisk in Fig. 2e, f). This phenotype was fully reproduced by EC-specific mCherry-dnTcf expression (Fig. 2f; Supplementary movies 9, 10). However, the time difference was not as prolonged in the latter as in IWR-1-treated embryos, presumably due to the turnover time of mCherry-dnTcf, as mCherry is no longer detectable at the endpoint of analysis.

In summary, our analysis reveals that Wnt signaling is required for anastomosis of brain capillaries.

### Block of Wnt signaling reduces VE-cadherin at cell junctions

**Fig. 3:**
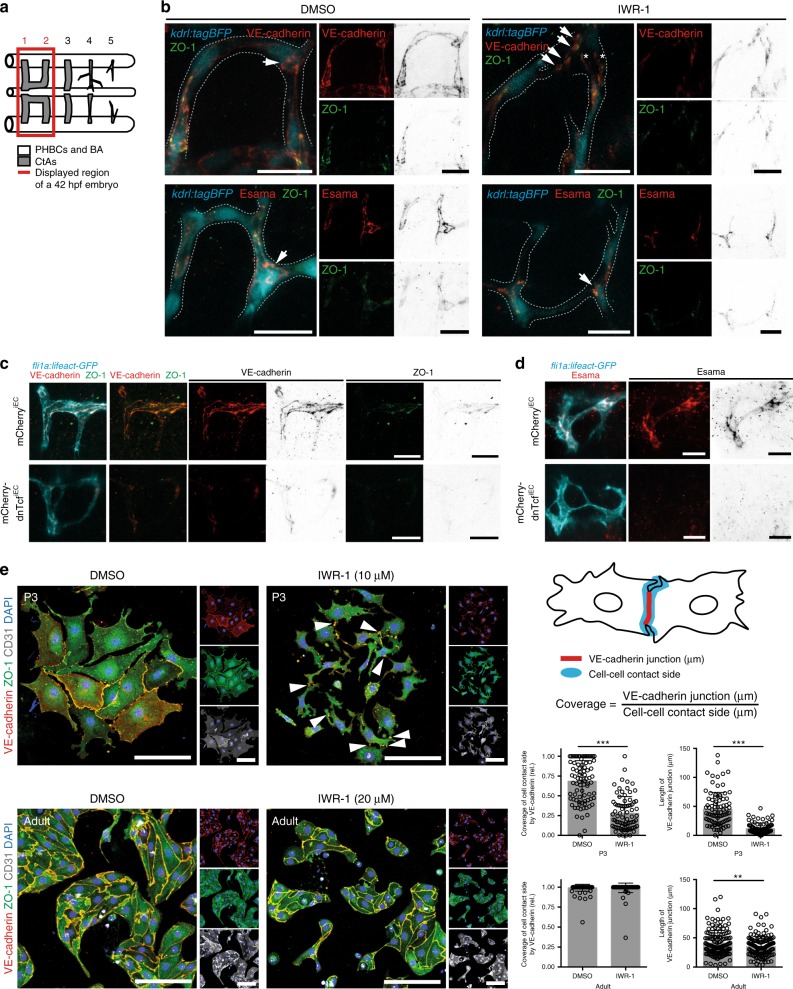
Junctional localization of VE-cadherin and Esama is affected by Wnt signaling inhibition. **a** Schematic of the zebrafish hindbrain vasculature at 42 hpf. Red box indicates region of analysis. In the following images (**b**–**d**) half of the boxed region is displayed. **b**–**d** Inhibition of Wnt signaling by IWR-1 from 29 hpf (**b**) or by dnTcf expression (**c**, **d**) strongly reduced expression of VE-cadherin or Esama and ZO-1 at the cell–cell junctions. Immunostaining for VE-cadherin (**b**, **c**; red) or Esama (**b**, **d**; red) and ZO-1 (**b**, **c**; green). Single channel images were displayed in inverted colors for better visualization. **b** In control embryos (DMSO), VE-cadherin and Esama were detected in cell–cell junctions along the CtAs and in anastomosis rings (arrow). Inhibition of Wnt signaling resulted in reduced staining of VE-cadherin and Esama at the cell junctions, formation of multiple small anastomosis rings (arrows) and ectopic VE-cadherin-positive cell protrusions (asterisks). **c**, **d** In control embryos (mCherry^iEC^), VE-cadherin and Esama are strongly expressed in cell–cell junctions. Expression of mCherry-dnTcf^iEC^ dramatically reduced VE-cadherin and ZO-1 (**c**) or Esama (**d**) at the cell–cell junctions. **e** Primary mouse BECs enriched from microvascular fragments from P3 mice exhibited impaired VE-cadherin junction formation following Wnt signaling inhibition by IWR-1 (DMSO: *n* = 86; IWR: *n* = 80; *N* = 4). In contrast, BECs isolated from adult mice had coverage of cell–cell contact sides by VE-cadherin similar to control (DMSO: *n* = 143; IWR-1: *n* = 130; *N* = 4). Immunostaining for VE-cadherin (red), ZO-1 (green), CD31 (white), and DAPI (blue) of cultured primary mouse BECs after treatment with IWR-1 (P3: 10 µM; adult: 20 µM) or DMSO, respectively. Values represent mean ± SD. *p < 0.05, **p < 0.01, ***p < 0.001, Student’s *t*-test; *n,* number of analyzed junctions; *N,* number of biological replicates. BECs, brain ECs, CtAs, central arteries; ECs, endothelial cells; Scale bars: 20 µm (**b**–**d**); 100 µm (**e**)

To elucidate how Wnt signaling regulates CtA anastomosis, we investigated the abundance of the EC-specific adhesion molecules VE-cadherin and Esama at cell–cell junctions of the CtAs^23,24^. VE-cadherin, a member of the cadherin protein family expressed in ECs, has been shown to be crucial for EC contact formation during blood vessel anastomosis^5,22^. Esam belongs to the protein family of Junction adhesion molecules (Jam) and two paralogues exist in zebrafish, *esama* and *esamb*, of which *esama* is mainly expressed in the vasculature^24^. To address, whether Wnt signaling inhibition affects protein localization of VE-cadherin or Esama, we performed immunostaining for VE-cadherin, Esama and ZO-1 in 42 hpf-old embryos (Fig. 3a–d). In control embryos (DMSO), VE-cadherin and Esama were expressed continuously along cell–cell junctions of ECs within CtAs and in anastomosis rings, which form at positions where two CtAs fuse (arrows in Fig. 3b). After IWR-1 treatment (Fig. 3b) or EC-specific mCherry-dnTcf expression (Fig. 3c, d), we found that the protein abundance of VE-cadherin, Esama, and ZO-1 at cell–cell junctions was dramatically reduced. Furthermore, we frequently observed clustered protein aggregates (stars in Fig. 3b) and the formation of many small adhesion spots (arrows in Fig. 3b) in Wnt-inhibited embryos, which is in line with our previous observations of multiple filopodia contacts (Fig. 2e, f). Hence, reduction and aberrant localization of VE-cadherin and Esama at the cell junctions could cause the anastomosis defects. To validate these findings in another model, we isolated primary brain endothelial cells (BECs) from microvascular fragments^34^ of either P3 or adult mice. We were able to block Wnt signaling using 10 µM IWR-1 for P3 and 20 µM for adult brain ECs (Fig. 3e, Supplementary Fig. 2e). To analyze the effect on VE-cadherin distribution we measured the length of the potential cell–cell contact area in form of opposing or touching membrane length and the actual coverage of that area by VE-cadherin. We observed a marked reduction in the length of VE-cadherin junctions and the coverage of cell–cell contact sides by VE-cadherin in ECs isolated from P3 mice. Interestingly, Wnt signaling inhibition did not affect junctional VE-cadherin in adult brain ECs. We conclude that brain capillary ECs from P3 mice are capable of responding to Wnt signaling, presumably because these brain ECs are in a more angiogenic state, whereas ECs from adult mice gain mature BBB properties and are no longer sensitive to Wnt signaling inhibition.

We next analyzed how a complete loss of VE-cadherin affects CtA lumen formation using homozygous *ve-cadherin*^*ubs8*^^35^ mutant embryos. In *ve-cadherin*^*ubs8*^-deficient mutants, CtAs form and invade similarly to wild-type siblings (Supplementary movies 11, 12), but fail to establish stable cell–cell contacts, continuously produce filopodia toward the ECs in neighboring CtAs or BA and fail to form continuous lumens (Supplementary Fig. 4a, b, Supplementary movies 11, 12), a behavior, which mimics our observations after Wnt signaling inhibition (Fig. 2e, f). Interestingly, intracellular signaling via VE-cadherin and its association with intracellular adaptor proteins seems to be required, as overexpression of VE-cadherin lacking the intracellular domain could not rescue the lumen formation defects (Supplementary Fig. 4d). Additionally, rescuing the *ve-cadherin*^*ubs8*^ mutant by expression of endogenous levels of the VE-cadherin tension sensor (VE-cad-TS)^36,37^ did not completely restore wild-type VE-cadherin function, (Supplementary Fig. 4a), indicating that the presence of the fluorophores within the intracellular domain of VE-cadherin reduces its signaling somewhat. We also analyzed lumen formation of CtAs in *esama*^*ubs19*^^24^ mutant embryos. Homozygous *esama*^*ubs19*^ mutants displayed fewer lumenized CtAs compared to heterozygous controls (Supplementary Fig. 4c). We conclude that VE-cadherin is required for CtA anastomosis and that Esama contributes to efficient CtA lumen formation, which is in agreement with previous reports^23,24^. Therefore, we reason that the lumen formation defects caused by Wnt signaling inhibition were the result from decreased VE-cadherin and Esama protein levels at the cell–cell contact sites in CtAs.

### Wnt does not regulate transcription of *ve-cadherin* and *esama*

**Fig. 4:**
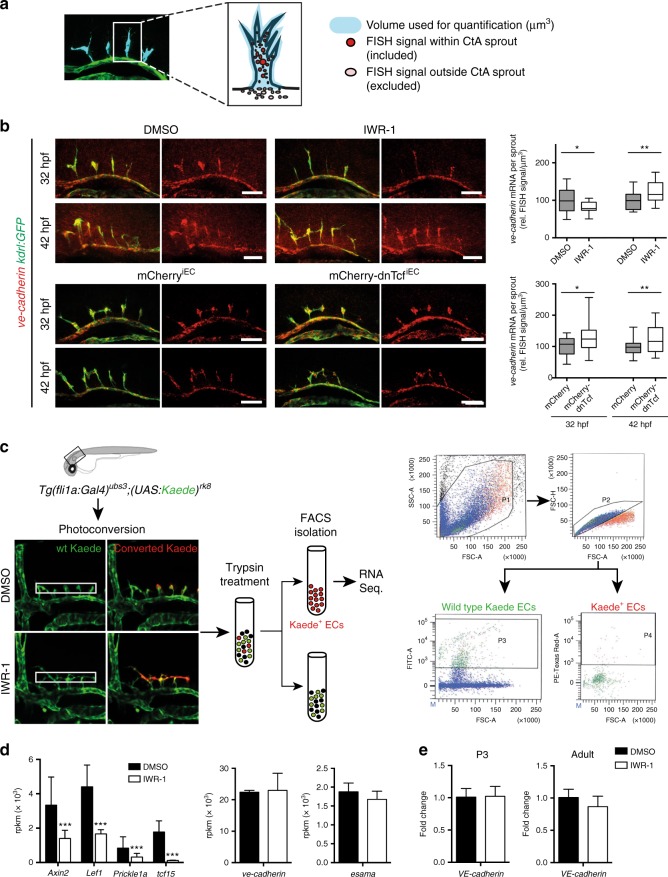
Wnt signaling does not regulate *ve-cadherin* mRNA expression. **a** Whole mount fluorescent in situ hybridization (FISH, red) in combination with anti-GFP immunostaining (green) of *Tg(kdrl:GFP)*^*s843*^ embryos. For quantification the FISH signal within CtA sprouts was normalized to the respective GFP volume (blue) of each sprout. **b** Inhibition of Wnt signaling either by incubation with IWR-1 or expression of mCherry-dnTcf^iEC^ did not severely affect *ve-cadherin* mRNA expression at 32 or 42 hpf. Treatment with IWR-1 resulted in slightly decreased *ve-cadherin* mRNA levels in CtA sprouts at 32 hpf, but in increased *ve-cadherin* mRNA levels at 42 hpf (32 hpf: DMSO: *n* = 16, *N* = 6, and IWR-1: *n* = 15, *N* = 6; 42 hpf: DMSO: *n* = 28, *N* = 7, and IWR-1: *n* = 19, *N* = 7). After mCherry-dnTcf^iEC^ expression in ECs *ve-cadherin* mRNA expression was slightly increased (32 hpf: mCherry^iEC^: *n* = 20, *N* = 6; mCherry-dnTcf^iEC^: *n* = 18, *N* = 7; 42 hpf: mCherry^iEC^: *n* = 40, *N* = 7; mCherry-dnTcf^iEC^: *n* = 16, *N* = 7). Quantifications were represented by Box-and-Whisker plots with median (center line), 25th and 75th percentiles (bounds of box) and Min-to-Max (whiskers), *p < 0.05, **p < 0.01, ***p < 0.001, Student’s *t*-test; *n,* number of CtA volumes analyzed; *N,* number of embryos analyzed. Scale bars: 50 µm. **c**, **d** Expression levels of *ve-cadherin* or *esama* were not altered by inhibition of Wnt signaling, whereas classical Wnt-target genes (e.g., *axin2*, *lef1*) were downregulated. For RNA sequencing, CtA ECs of *Tg(fli1a:Gal4);(UAS:Kaede)* embryos, which were treated with IWR-1 or DMSO, were photoconverted using the confocal microscope laser and isolated by FACS (photoconverted Kaede ECs = Kaede^+^ cells = P4) (*n* = 3). **e** Expression levels of *VE-cadherin* are not affected by IWR-1 treatment. RT-qPCR analysis of Values in **d**, **e** represent mean ± SD, *p < 0.05, **p < 0.01, ***p < 0.001, *n,* number of biological replicates. BECs, brain ECs, CtAs, central arteries; ECs, endothelial cells; FISH, fluorescent in situ hybridization

We hypothesized that the reduced protein levels of VE-cadherin and Esama could result from reduced transcription in embryos lacking Wnt signaling. In order to analyze *ve-cadherin* and *esama* mRNA expression levels, we performed whole mount fluorescent in situ hybridization (FISH) after Wnt signaling inhibition by IWR-1 or EC-specific mCherry-dnTcf expression (Fig. 4a, b, Supplementary Fig. 4e). We quantified the fluorescence intensity within CtAs at 32 or 42 hpf and normalized the fluorescence intensity to the respective CtA volume (generated from *Tg(kdrl:GFP)*^*s843*^ signal). Surprisingly, we did not find severe changes of the overall *ve-cadherin* or *esama* mRNA abundance in CtA ECs at 32 or 42 hpf (Fig. 4a, b, Supplementary Fig. 4e).

At 32 hpf, *ve-cadherin* mRNA was slightly decreased following IWR-1 treatment and slightly increased following mCherry-dnTcf^iEC^ expression. At 42 hpf, at which we detected reduced VE-cadherin protein levels at the cell–cell junctions (Fig. 4b), *ve-cadherin* mRNA was increased using either of the Wnt signaling inhibition approaches. This indicates, that *ve-cadherin* mRNA levels in CtAs were not decreased (as expected from a putative Wnt target gene), but rather slightly increased over time, presumably due to a compensatory mechanism. The mRNA level of *esama* in CtA ECs was slightly decreased after Wnt signaling inhibition by IWR-1 or mCherry-dnTcf expression both at 32 and 42 hpf (Fig. S4e).

Additionally, to gain insights into transcriptional changes in CtAs mediated by Wnt signaling inhibition, we isolated Kaede-photoconverted CtA ECs from 1.5 days-old *Tg(fli1a:Gal4)*^*ubs3*^*;(UAS:Kaede)*^*rk8*^ embryos, which were treated with IWR-1 or DMSO (Fig. 4c, d) and performed gene expression profiling by RNA sequencing. We confirmed that *ve-cadherin* and *esama* expression levels were not regulated by Wnt signaling in sharp contrast to classical Wnt target genes (e.g., *axin2* and *lef1*, Fig. 4d). We also analyzed the effect of Wnt inhibition on *VE-cadherin* mRNA levels by qPCR in BECs isolated from P3 and adult animals. In line with our zebrafish data, we observed no transcriptional regulation of mouse *VE-cadherin* by Wnt signaling (Fig. 4e).

We therefore conclude that post-transcriptional mechanisms account for the changes in protein abundance at brain capillary cell–cell junctions following Wnt signaling inhibition.

### Wnt signaling counteracts S1pr1 signaling in angiogenesis

To address how VE-cadherin localization at cell–cell junctions can be regulated during brain angiogenesis, we focused on Sphingosine-1-phosphate (S1p) signaling, as it is known to regulate EC integrity and behavior by modulating junction protein localization and cell contractility^27,28^. In particular, S1p-induced signaling mainly via S1p receptor 1 (S1pr1) has been shown in vivo and in vitro to increase recruitment of Cadherin protein family members, including VE-cadherin and N-cadherin, to the plasma membrane^31,38,39^. Additionally, S1p receptor 3 (S1pr3) was found to function cooperatively with S1pr1 in promoting adherens junction formation in ECs^38^, whereas S1p receptor 2 (S1pr2) signaling disrupted EC junctions, thereby increasing vascular permeability^40^. We confirmed that similar to what is seen in mice^30^, blocking of S1prs using the pharmacological antagonist VPC23019 (VPC) during barriergenesis (48–72 hpf) resulted in extravasation of red blood cells into the surrounding brain tissue, indicating breakdown of the BBB (Supplementary Fig. 5a).

Our RNA sequencing data showed expression of *s1pr1* and *s1pr5a* as well as very low level expression of *s1pr2* and *s1pr3b* in zebrafish brain capillary ECs (Supplementary Fig. 5b). In order to functionally dissect the contribution of these different S1pr’s we validated various pharmacological antagonists by their potency to rescue phenotypes of S1pr overexpression (Supplementary Fig. 5c–g). In the zebrafish, VPC seems to antagonize the tested S1pr’s (S1pr1, S1pr2, S1pr3a, and S1pr5a), whereas TY52156 (TY) acts specifically on S1pr1. In our hands the S1pr2 antagonist JTE013 (JTE) did not rescue S1pr2 overexpression (Supplementary Fig. 5c–g).

**Fig. 5:**
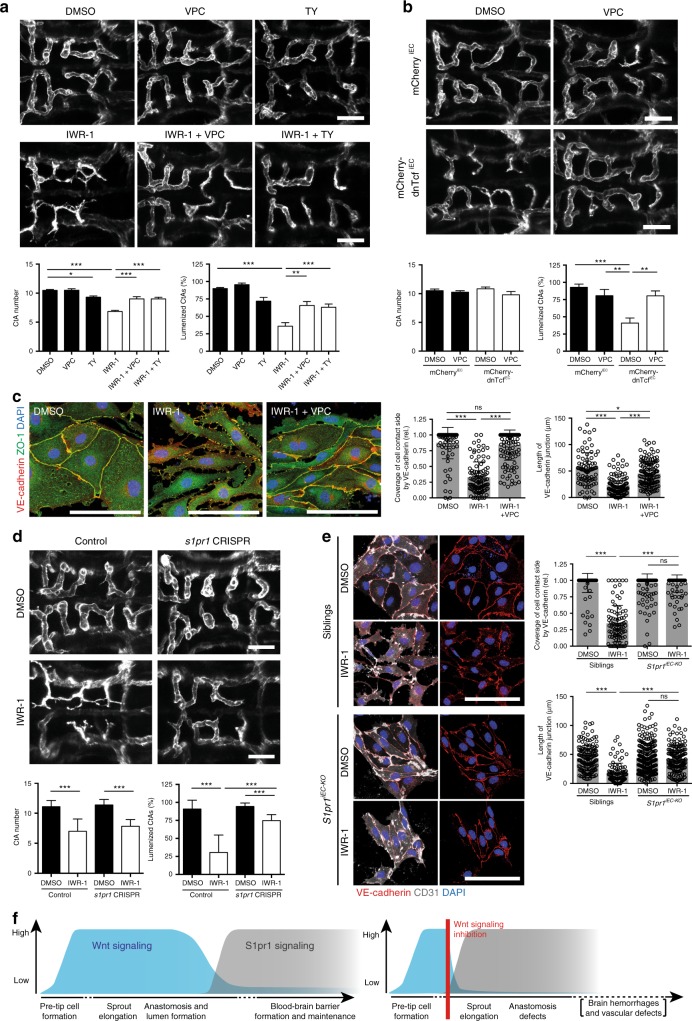
Wnt signaling counteracts S1pr1 signaling during brain capillary angiogenesis. **a** Treatment with Sphingosine-1-phoshate receptor (S1pr) antagonists from 29 to 48 hpf did not affect CtA angiogenesis. Combining inhibition of Wnt signaling with antagonizing S1pr signaling led to a rescue of CtA angiogenesis and lumen formation. Embryos were single or co-treated with IWR-1 and S1pr antagonists VPC23019 (VPC), and TY52156 (TY) (DMSO: *n* = 23; VPC: *n* = 8; TY: *n* = 10; IWR-1: *n* = 33; IWR-1 + VPC: *n* = 10; IWR-1 + TY: *n* = 15). **b** Antagonizing S1pr signaling following EC-specific dnTcf expression rescues CtA lumen formation defects. Embryos expressing mCherry^iEC^ or mCherry-dnTcf^iEC^ were treated with VPC following heat shock at 26 hpf. (mCherry^iEC^: DMSO: *n* = 4, VPC: *n* = 4; mCherry-dnTcf^iEC^: DMSO: *n* = 6, VPC: *n* = 5). **c** Co-treatment with VPC restored the VE-cadherin junction formation defects of IWR-1-treated primary mouse BECs (from P3 animals; DMSO: *n* = 84; IWR-1: *n* = 100; IWR-1 + VPC: *n* = 134; *N* = 3). **d** Transient CRISPR-Cas9-mediated knockout of *s1pr1* resulted partial rescue of the lumen formation defects in Wnt-depleted embryos. Embryos were injected with gRNA targeting *s1pr1* and *cas9* mRNA (*s1pr1* CRISPR) or *cas9* mRNA (control) and treated with IWR-1 from 29 to 48 hpf (control: DMSO: *n* = 11, IWR-1: *n* = 12; s1pr1 CRISPR: DMSO: *n* = 8, IWR-1: *n* = 12). **e** EC-specific knockout of S1pr1 (*S1pr1*^*iEC-KO*^) caused insensitivity to IWR-1 in mouse primary BECs. BECs isolated from *S1pr1*^*iEC-KO*^ animals at P3 showed no difference in VE-cadherin junction formation after DMSO or IWR-1 treatment in contrast to control siblings (siblings: DMSO: *n* = 152; IWR: *n* = 92; *N* = 4; *S1pr1*^*iEC-KO*^: DMSO: *n* = 240; IWR: *n* = 156; *N* = 5). **f** Illustration of Wnt and S1pr1 signaling during brain vascularization in wild type and Wnt-depleted embryos. Confocal images of zebrafish embryos (**a**, **b**, **d**) show dorsal views (anterior to the left) and GFP expression by *Tg(fli1a:lifeact-GFP)*^*mu240*^. Images of mouse BECs (**c**, **e**) display immunostaining for VE-cadherin (red), ZO-1 (green), and DAPI (blue). Values represent mean ± SEM (**a**, **b**) or mean ± SD (**c**–**e**). *p < 0.05, **p < 0.01, ***p < 0.001, One-way ANOVA; *n,* number of analyzed embryos (**a**, **b**, **d**); *n,* number of analyzed junctions, *N,* number of biological replicates (**c**, **e**); BECs, brain ECs, CtAs, central arteries; ECs, endothelial cells; ns, not significant; S1pr, sphingosine-1-phosphate receptor; Scale bars: **a**, **b**, **d** = 50 µm; **c**, **e** = 100 µm

To test the effects of S1pr inhibition on lumen formation and VE-cadherin localization, we next applied the antagonists of S1pr signaling during CtA angiogenesis at 29 hpf and assessed patterning and lumen formation at 48 hpf. In contrast to barriergenesis, treatment during brain angiogenesis with either VPC or TY did not affect CtA development and lumen formation in zebrafish (Fig. 5a). It is therefore likely that in wild-type embryos S1pr signaling is required only after the onset of blood circulation in CtAs, presumably through delivery of high amounts of its ligand S1p by the blood^41^. To our surprise, co-treatment of embryos with the Wnt signaling inhibitor IWR-1 and either VPC or TY increased the proportion of lumenized CtAs compared to single IWR-1 treatment and rescued CtA patterning and lumen formation (Fig. 5a). S1pr inhibition by VPC was also able to rescue EC-specific loss of Wnt signaling in embryos overexpressing mCherry-dnTcf^iEC^ (Fig. 5b).

We further analyzed, whether not only lumen formation, but also VE-cadherin and Esama levels at cell–cell junctions were rescued by inhibiting S1pr signaling in Wnt-depleted embryos. We observed that the reduced VE-cadherin and Esama localization at cell–cell contact sides and in anastomosis rings after IWR-1 treatment was restored by co-treatment with the S1pr1 antagonist TY and similar to control embryos when analyzed either by immunostaining for VE-cadherin and Esama or by live imaging of the Venus-fluorophore distribution of the VE-cadherin-TS (Supplementary Fig. 6). Hence, blocking of S1pr1 in parallel to IWR-1 treatment was sufficient to rescue VE-cadherin and Esama protein levels at cell–cell junctions. In line with these results, inhibition of S1pr signaling by VPC restored VE-cadherin-positive junctions in IWR-1 treated P3 BECs (Fig. 5c).

As an alternative approach to pharmacological S1pr1 inhibition in zebrafish, we used transient CRISPR-Cas9-mediated *s1pr1* knockdown in combination with IWR-1 treatment. This manipulation restored lumen formation of CtAs (Fig. 5d) similar to VPC or TY treatment (Fig. 5a). Furthermore, Wnt signaling inhibition was unable to affect VE-cadherin localization in *S1pr1*^*iEC*^ knockout BECs isolated from P3 mice (Fig. 5e).

Therefore, loss of S1pr1 signaling rescued Wnt depletion phenotypes, such as impaired lumen formation and VE-cadherin localization. Taken together, the studies reveal that Wnt signaling regulates brain capillary angiogenesis by counteracting S1pr1 signaling in zebrafish and mouse ECs, presumably to prevent premature barrier formation, which would impair brain angiogenesis (Fig. 5f).

### Wnt signaling prevents S1pr1-mediated Rac1 activation

**Fig. 6:**
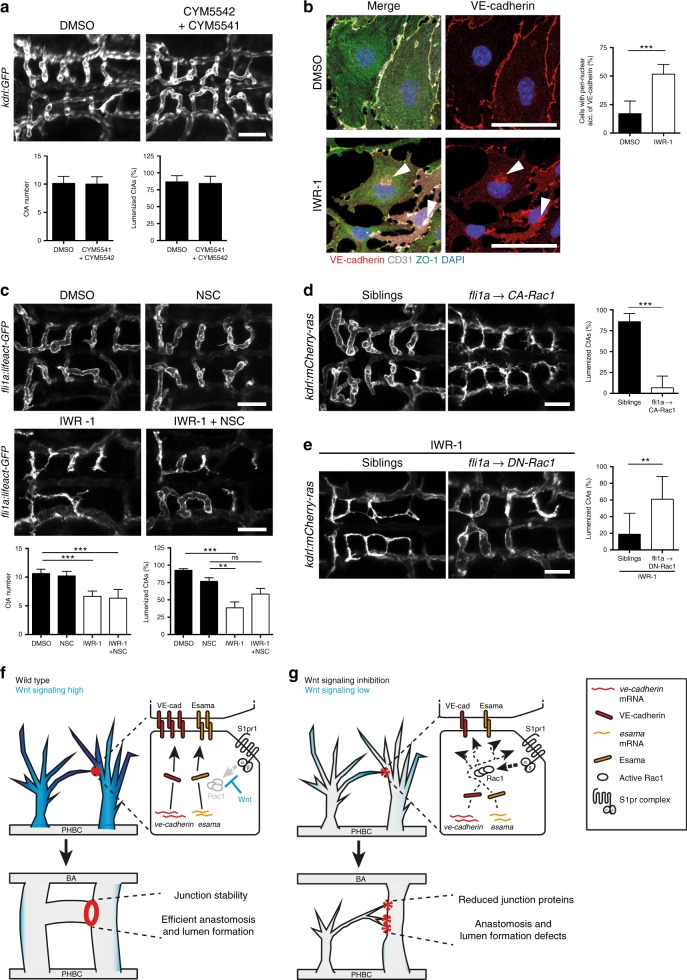
Wnt signaling regulates S1pr1 signaling downstream of receptor activation. **a** Treatment of zebrafish embryos with S1pr agonists did not result in CtA formation or anastomosis defects. Wild-type embryos were treated from 29 to 48 hpf with S1pr agonists (CYM5541 and CYM5542) or DMSO (DMSO: *n* = 10; agonists: *n* = 10). **b** High resolution imaging of IWR-1-treated mouse primary BECs revealed intracellular accumulation of VE-cadherin. BECs isolated from P3 animals were treated with IWR-1 or DMSO (DMSO: *n* = 12; IWR-1: *n* = 12; *N* = 4). **c** Inhibition of Rac1 rescued lumen formation defects of CtAs in Wnt-depleted embryos. Embryos were single or co-treated with IWR-1 and NSC23766 (NSC, Rac1 inhibitor) from 29 to 48 hpf. (DMSO: *n* = 8; NSC: *n* = 10; IWR-1: *n* = 14; IWR-1 + NSC: *n* = 12). **d** EC-specific overexpression of CA-Rac1 results in lumen formation defects. Analysis of *Tg(fli1a:Gal4)*^*ubs3*^*;(UAS:CA-Rac1)*^*mu211*^ embryos and control siblings at 48 hpf (siblings: *n* = 15, CA-Rac1: *n* = 17). **e** EC-specific overexpression of DN-Rac1 partially rescues IWR-1-induced lumen formation defects. Treatment of *Tg(fli1a:Gal4)*^*ubs3*^*;(UAS:DN-Rac1)*^*mu212*^ embryos and control siblings with IWR-1 from 29 to 48 hpf (siblings: *n* = 17, DN-Rac1: *n* = 5). **f**, **g** Molecular events of brain capillary anastomosis in wild type (**f**) and Wnt-depleted (**g**) embryos. Wnt signaling is highly active in wild-type brain capillary sprouts (**f**, dark blue), which allows for efficient localization of VE-cadherin (red) and Esama (yellow) to cell–cell junctions, thus resulting in rapid anastomosis and lumen formation. Following Wnt signaling inhibition (**g**), brain capillary anastomosis is compromised due to VE-cadherin and Esama mislocalization caused by premature S1pr1 signaling, which involves activation of Rac1. Confocal images of zebrafish embryos (**a**, **c**, **d**, **e**) show dorsal views (anterior to the left) and EC-labeling GFP or mCherry as indicated. Images of mouse BECs (**b**) display immunostaining for VE-cadherin (red), CD31 (white), ZO-1 (green), and DAPI (blue). All values represent mean ± SD. *p < 0.05, **p < 0.01, ***p < 0.001, One-way ANOVA; *n* number of analyzed embryos (**a**, **c**, **d**, **e**); *n,* number of analyzed embryos, *N,* number of biological replicates (**b**); BBB, blood-brain barrier, BECs, brain ECs, CtAs, central arteries; ECs, endothelial cells; ns, not significant; S1pr, sphingosine-1-phosphate receptor; Scale bars: **a**, **c**, **d**, **e** = 50 µm; **b** = 100 µm

So far we have shown that Wnt signaling regulates brain capillary angiogenesis in an EC-specific manner by post-transcriptionally influencing VE-cadherin localization. Furthermore, the mislocalization of VE-cadherin caused by Wnt signaling inhibition was dependent on S1pr1 signaling (Fig. 5, Supplementary Fig. 6). In order to understand the regulatory interaction of these two signaling pathways, we addressed at which level Wnt signaling interferes with S1pr1 signaling activation. We therefore stimulated S1pr1 signaling using a combination of two agonists (CYM5541 and CYM5542; validation for agonist activity, see Supplementary Fig. 5h). Overstimulation of S1pr1 signaling activity did not induce lumen formation defects (Fig. 6a) and therefore did not overcome the intrinsic counteraction by Wnt signaling. However, block of S1pr signaling in *ve-cadherin* or *esama*-deficient embryos did not result in a rescue of lumen formation defects (Supplementary Fig. 7a). Therefore, Wnt signaling antagonizes S1pr1 signaling downstream of receptor activation, but upstream of VE-cadherin and Esama function.

One potential mechanism that could account for the downregulation of VE-cadherin could be the regulation of VE-cadherin degradation or internalization. We therefore inhibited proteasomal degradation by MG132, dynamin-dependent internalization by Dynasore and lysosomal degradation by Chloroquine; however, none of these treatments rescued Wnt-deficiency (Supplementary Fig. 7b, c). Interestingly, we also observed a mislocalization of VE-cadherin to seemingly intracellular vesicular structures in Wnt signaling-deficient BECs (Fig. 6b), indicating changes in cellular architecture rather than VE-cadherin degradation following Wnt signaling inhibition.

S1p-induced signaling in cultured ECs has been linked to activation of the Rho/Rac-pathway for stabilization of the cytoskeleton and recruitment of junction proteins to the cell membrane^38,42,43^. Similar to S1pr signaling, Rac1 activation as well as Rho inactivation is known to promote EC barrier function in mature blood vessels^44^; however, their contribution to brain capillary angiogenesis has not been addressed yet. We therefore applied the pharmacological Rac1 inhibitor (NSC23766, NSC) or Rho inhibitor (CT03) alone or together with IWR-1 as described. Single treatment with either Rac1 or Rho inhibitors, did not affect CtA development or lumen formation. Indeed, co-treatment of IWR-1 and Rac1 inhibitor, but not of IWR-1 and Rho inhibitor, robustly restored the lumen formation defects caused by IWR-1 (Fig. 6c, Supplementary Fig. 8a).

We hypothesized that if Rac1 was mediating premature S1pr1 signaling activation, overexpression of constitutive-active Rac1 (CA-Rac1) should phenocopy Wnt signaling inhibition. Therefore, we mated *Tg(fli1a:Gal4)*^*ubs3*^ fish with *Tg(UAS:CA-Rac1)*^*mu211*^ and analyzed brain capillary lumen formation in 48 hpf-old embryos (Fig. 6d). Vascular-specific overexpression of CA-Rac1 caused strong lumen formation defects and was accompanied by multiple filopodia contact sides comparable to IWR-1-treated (Fig. 2a) or *ve-cadherin*-deficient (Supplementary Fig. 4a) embryos. Additionally, vascular-specific Gal4-UAS-mediated overexpression of dominant negative Rac1 (DN-Rac1, from *Tg(UAS:DN-Rac1)*^*mu212*^) could partially rescued lumen formation in IWR-1-treated embryos (Fig. 6e).

Hence, Rac1 activation downstream of S1pr1 signaling is required and sufficient to mediate lumen formation defects caused by Wnt signaling inhibition.

In summary, we identified a temporal-specific function of Wnt signaling for brain capillary angiogenesis by post-transcriptionally and EC-specifically regulating junction protein localization upstream or at the level of Rac1 activity, but downstream of S1pr1 signaling. We suggest that EC-specific Wnt signaling counteracts S1pr1 signaling during CtA angiogenesis, presumably to prevent premature barrier formation processes, which would impair brain angiogenesis (Fig. 6f,g).

Interestingly this mechanism seems to be specific to the role of Wnt signaling in regulating anastomosis and lumen formation, but not to the essential function of Wnt signaling during the earlier pre-tip cell phase^12,15^. Treatment of embryos lacking Wnt signaling following IWR-1 treatment or of *gpr124* mutants with S1pr antagonists did not restore CtA sprout formation (Supplementary Fig. 8b, c).

In conclusion, we describe a temporal control of different steps of brain angiogenesis and BBB formation, which are differentially regulated by Wnt signaling. We also discover an interplay between Wnt and S1pr signaling during brain angiogenesis.

## Discussion

We addressed the role of β-catenin-dependent Wnt signaling during early brain capillary angiogenesis in vivo. We found that (1) Wnt signaling is highly active in brain capillary sprouts, but after pre-tip cell formation it is dispensable for brain capillary invasion into the brain parenchyma. Instead, (2) Wnt signaling is required for brain capillary anastomosis. During this process, (3) Wnt signaling influences the post-transcriptional regulation and cell–cell junction localization of the EC-specific adhesion molecules, VE-cadherin, and Esama, by counteracting S1pr1 signaling (Fig. 6f, g).

We and others have shown that Wnt signaling is active in brain capillaries during brain angiogenesis (Fig. 1,^11,12,14^). Recent reports demonstrate that Gpr124-Reck-mediated Wnt signaling is essential for brain vascularization, as homozygous *grp124* or *reck* mutants are completely devoid of brain capillaries^12,15–17^. We hypothesized that Wnt signaling might be involved in the formation of pre-tip cells within the parental vessel, which most likely occurs at earlier developmental stages^12^. In line with this, a role of Wnt signaling for EC specification has already been suggested to drive cell fate from the Fli1a-positive precursor cell population into ECs at the expense of primitive erythrocytes^33^. At later developmental stages, Wnt signaling is required for subspecification of EC populations within the vasculature, which has been shown for hemogenic EC formation^45,46^ and lymphatic cell fate specification^47^.

Here we demonstrate that Wnt signaling is continuously activated in CtAs during all stages of angiogenesis. Surprisingly, Wnt signaling is not involved in the process of CtA sprout invasion and filopodia formation (Fig. 1).

However, our data demonstrate that Wnt signaling is essential for brain capillary anastomosis (Fig. 2). During the anastomosis process in intersegmental vessels, VE-cadherin is required for cell–cell contact formation and together with Esama has been shown to be essential for efficient blood vessel fusion^23,24^. We detected reduced protein levels of VE-cadherin, Esama, and ZO-1 at the cell–cell junctions following Wnt signaling inhibition (Fig. 3), which presumably impair anastomosis and lumen formation. In line with these findings, knockouts of VE-cadherin (by *ve-cadherin*^*ubs8/ubs8*^) or Esama (by *esama*^*ubs19/ubs19*^) phenocopied CtA lumen formation defects (Supplementary Fig. 4). Surprisingly, neither *ve-cadherin* nor *esama* are transcriptional targets of Wnt signaling (Fig. 4). Similar to Hupe and colleagues, we detected mildly increased amounts *ve-cadherin* mRNA when Wnt signaling inhibition persists for longer time intervals (>12 h), indicating a compensatory response to the reduced protein levels of VE-cadherin at cell–cell junctions^48^. We therefore conclude that VE-cadherin and Esama downregulation occurs via a post-transcriptional mechanism, which interferes with their membrane localization. Using a series of inhibitors, we excluded internalization as well as proteasomal and lysosomal degradation as potential mechanisms (Supplementary Fig. 7). Previously, Wnt signaling has been shown to be required for the junction localization of Claudin 1 and 3 in murine brain ECs during BBB formation, but no direct transcriptional regulation has been detected^13,14^. As there have been no mechanisms proposed of how Wnt signaling could regulate these junctional molecules, we speculate that they might also be downregulated post-transcriptionally as a consequence of imbalanced S1pr signaling.

The concept that S1pr signaling regulates EC integrity by modulating translocation of junction proteins and actomyosin contractility is widely accepted^27,28^. So far, S1pr signaling has not been linked to brain capillary angiogenesis and our data also indicate that the endogenous S1pr signaling is neither required for CtA tip cell specification (Supplementary Fig. 5) nor for other aspects of CtA angiogenesis (Fig. 5). However, as recently shown in mice^30^, we demonstrate that S1pr signaling indeed regulates BBB formation and integrity in zebrafish (Supplementary Fig. 5). Moreover, our data points to a novel regulatory mechanism of Wnt signaling during brain capillary angiogenesis, which is to counteract S1pr-induced BBB formation in order to ensure efficient anastomosis (Fig. 5). We propose that Wnt signaling suppresses S1pr1-mediated signaling in CtAs during early stages of brain vascularization. After lumen formation, Wnt signaling activity within brain capillaries decreases to a low baseline level (Fig. 1^14^). In lumenized vessels, activation of S1pr signaling would occur and be most likely enhanced by increased supply of the ligand S1p through the blood circulation^41^, which would enable functional S1pr signaling for BBB formation and maintenance (Supplementary Fig. 5).

It seems likely that the S1pr1-mediated pathway signals via G_αi_ and activates Rac1 (Fig. 6^27,49^). We hypothesize that over-activation of Rac1 after Wnt signaling inhibition interferes with efficient cellular junction formation during brain capillary angiogenesis. To date, Rac1 activation is well described to promote EC barrier formation in mature blood vessel^44^, indicating that Rac1 can act downstream of S1pr signaling in barriergenesis^27^. However, we demonstrate a different regulation in Wnt-responsive brain capillary ECs during capillary angiogenesis, which is similar to our observation of S1pr signaling regulation (Fig. 5). Our data in zebrafish and mouse BECs clearly show altered responsiveness of the ECs to Wnt inhibition depending on their developmental state. This indicates that S1pr-Rac1-mediated regulation of VE-cadherin is differently affecting the modulation of established junctions in mature blood vessels compared to junctions newly forming during brain angiogenesis.

Additionally, over-activation of Rac1 could destabilize junction molecule complexes, as has been shown for initial stages of pharyngeal pouch formation in zebrafish^50^. In this study, the authors suggested that Wnt signaling mediates activation of Rac1 by an unknown mechanism^50^; however, a possible role of S1pr signaling has not been addressed. The authors also observed a reduced membrane localization of the adherens junction molecules E-Cadherin and Alcama after Wnt signaling inhibition by morpholino-mediated knockdown of Disheveled, which is in line with our findings during brain angiogenesis. We therefore speculate that similarly to brain capillary ECs, Wnt signaling regulates Rac1-mediated S1pr signaling and thereby modulate junction localization of adhesion molecules also during pharyngeal pouch formation.

During brain neo-vascularization, we and others report divers functions of Wnt signaling, which regulate many steps of brain capillary angiogenesis and BBB formation and cross-communicate with other signaling cues^51^. First, Wnt signaling is required for the formation of the brain capillary pre-tip cells within the parental vessel, which is dependent on a functional Gpr124-Reck co-receptor complex^12,15–18^. Second, Wnt signaling limits S1pr signaling during brain capillary sprouting and promotes brain capillary anastomosis through efficient VE-cadherin and Esama localization to the membrane (as shown in this work). And third, Wnt signaling leads to the induction of BBB components, such as glucose transporter Glut1 and Claudin family members, already during brain angiogenesis in order to subsequently establish a functional BBB after brain angiogenesis has been completed^10,26,48,52^ and sealing of the BBB is likely to be promoted by S1pr signaling after the onset of blood circulation (Supplementary Fig. 5^30,41^). The complexity of the signaling network regulating EC behavior during brain angiogenesis and BBB formation is far from being understood. Our study provides a regulatory function of Wnt signaling during brain angiogenesis, which was only unraveled by interfering with Wnt signaling in a temporally and spatially restricted manner in combination with in vivo time-lapse imaging. We were able to identify a post-transcriptional level of regulation, which is essential for cell–cell junction formation and the modulation of EC barrier establishment or tightness. Therefore, interfering with Wnt or S1p signaling during treatment of infectious and neurodegenerative diseases as well as anti-cancer therapy remains a major clinical obstacle as adverse effects and cross-reactions are not well predictable^30,53–55^. The FDA approved sphingosine analog and agonist of S1pr1 and S1pr3–5 called Fingolimod (FTY720/Gilenya) has been reported to cause side effects of increased of vascular permeability in the lung and cardio-vascular complications. Moreover, cases with neuroencephalitis were noted when the drug was applied at higher doses. Therefore, the evaluation of the potential clinical impact for CNS development and pathology of these and future substances requires a greater understanding of signaling cues regulating EC properties and behavior.

## Methods

### Zebrafish husbandry and strains

Zebrafish (*Danio rerio*) husbandry and embryo maintenance were carried out under standard conditions at 28.5 °C^56^. Embryonic developmental stages were determined according to ref. ^57^.

Transgenic lines used in this study were *Tg(14TCF:loxP-STOP-loxP-dGFP)*^*mu202*^^33^, *Tg(axin2*_*BAC*_*:Venus-Pest)*^*mu288*^^33^, *Tg(fli1a:dsRed)*^*um13*^^58^, *Tg(fli1a:lifeact-GFP)*^*mu240*^^59^, *Tg(fli1a:nGFP)*^*y7*^^60^, *Tg(fli1ep:Gal4ff)*^*ubs3*^^61^, *Tg(gata1:dsRed)*^*sd2*^^62^, *Tg(kdrl:cre)*^*s898*^^63^, *Tg(kdrl:GFP)*^*s843*^^64^, *Tg(kdrl:mKate2-CAAX)*^*ubs16*^^23^, *Tg(kdrl:Hras-mCherry)*^*s896*^^65^, *Tg(kdrl:tagBFP)*^*mu293*^^66^, *Tg(UAS:Kaede)*^*rk8*^^67^, *Tg(UAS:VE-cadherinΔC-EGFP)*^*ubs12*^^23^ and *Tg(ve-cad*_*BAC*_*:ve-cadTS)*^*uq11bh*^ (referred as VE-cadTS)^36^. Mutants used in this study were *esama*^*ubs19*^^24^, *gpr124*^*s984*^^12^ and *ve-cadherin*^*ubs8*^^35^.

The *Tg(UAS:CA-Rac1)*^*mu211*^ and *Tg(UAS:DN-Rac1)*^*mu212*^ lines were generated by injection of plasmids, which were a gift from Gage Crump^50^, and according to standard transgenesis protocol^68^. Both constructs harbor *α-crystallin:cerulean*, inducing blue fluorescence in the lenses at 48 hpf. To overexpress CA-Rac1 and DN-Rac1 in the vasculature the respective fish were mated to *Tg(fli1ep:Gal4ff)*^*ubs3*^*;(kdrl:Hras-mCherry)*^*s896*^. Please note, that in the case of DN-Rac1 overexpression, all embryos were presorted for established circulation in PHBC and BA prior to analysis by confocal microscopy as we most likely obtained multiple integrations of the construct and thereby varying phenotypes in the offspring embryos.

### Generation of transgenic Wnt-manipulating lines

The *Tg(hsp70l:loxP-STOP-loxP-mCherry-dntcf)*^*mu201*^ line was generated by fusing the coding sequences of *mCherry*^69^ in frame with *dntcf*^70^ downstream of a heat shock inducible promoter (hsp70l^69^). A floxed STOP cassette (loxP-STOP-loxP, Hesselson 2009) was inserted upstream of *mCherry* by AflII-SpeI digest. Using the gateway system, *hsp70l:loxP-STOP-loxP-mCherry-dntcf* was cloned into the destination vector pTol2Dest^71^.

The *Tg(hsp70l:loxP-STOP-loxP-mCherry)*^*mu279*^ line was generated by removing the *dntcf* sequence from *Tol2-hsp70l:loxP-STOP-loxP-mCherry-dntcf-Tol2* using AfeI-SnaBI digest followed by Klenow blunting and religation.

For both lines, standard transgenesis was performed^68^.

### Microinjections

For ubiquitous removal of the floxed STOP cassette by Cre recombinase, 2 nL of *cre* mRNA (200 pg/nL) were injected at single cell stage. Therefore the *cre* coding sequence^69^ was cloned into pCS2+ and transcribed into mRNA by using the Sp6 mMessage mMachine kit (Ambion) following NotI-digest as previously described^33^.

For overexpression of S1pr’s, the coding sequences of *s1pr1*, *s1pr2*, *s1pr3a*, and *s1pr5a* were amplified from 24 hpf-cDNA and cloned into pCS2+ using the following primers (see also Supplementary Table 1): *s1pr1* fwd 5′-ATGGATGACCTAATCGCC-3′, *s1pr1* rev 5′-CGAGACGAAAAAGTTCACG-3′, *s1pr2* fwd 5′-ATGACTACTTGCCGTCTG-3′, *s1pr2* rev 5′-GGGATCTGCAAACACTTGG-3′, *s1pr3a* fwd 5′-ATGGATGACGAGCTTGAACC-3′, *s1pr3a* rev 5′-TCAGAACTTCCCCAAAGCG-3′, *s1pr5a* fwd 5′-GGTCAGCAGAAGTGAAATGG-3′, *s1pr5a* rev 5′-CAGACTTGTTTACTTGGCAG-3′. Transcription into mRNA following NotI-digest was carried out using the Sp6 mMessage mMachine kit (Ambion) as described^33^. Thereafter, 2 nL of 75 ng/µL or 150 ng/µL *s1pr1* mRNA, 10 ng/µL *s1pr2* mRNA, 150 ng/µL *s1pr3a* or 150 ng/µL *s1pr5a* mRNA were injected at single cell stage as indicated for each experiment. For agonist experiments, 75 ng/µL s1pr1 mRNA was additionally supplemented with 200 µM Sphingosine-1-phospate (S1p, Tocris Bioscience, 1370).

For transient CRISPR-Cas9-mediated knockout of *s1pr1*, annealed template oligonucleotides were transcribed into gRNAs using MEGAshortscript T7 Kit (Ambion)**:** *s1pr1* 5′- AAAGCACCGACTCGGTGCCACTTTTTCAAGTTGATAACGGACTAGCCTTATTTTAACTTGCTATTTCTAGCTCTAAAACTCTCTGAAGAACCATTGCGTTATAGTGAGTCGTATTACGC-3′ (*s1pr1* target sequence: 5′-TCTCTGAAGAACCATTGCGT-3′). A total of 2 nL of 500 ng/µL gRNA and 300 ng/µL *nls-zCas9-nls* mRNA^72^ or only *nls-zCas9-nls* mRNA as control were injected at single cell stage. Efficacy of the CRISPR-targeting was accessed by PCR using *s1pr1* fwd 5′-TCTATACTGCCAACATCCTG-3′ and rev 5′-CTGATGAGCATGAAGACCC-3′ (see also Supplementary Table 1) and subsequent restriction enzyme digest using Hpy188I. This leads to 136 bp and 69 bp fragments in control samples and additionally ~205 bp fragments in samples obtained from *s1pr1* CRISPR injected embryos.

### Vascular-specific Cre-mediated recombination

For vascular-specific removal of floxed STOP-cassettes, transgenic fish (e.g. *Tg(hsp70l:loxP-STOP-loxP-mCherry-dntcf)*^*mu201*^) were mated with *Tg(kdrl:cre)*^*s898*^. The resulting embryos carrying both transgenes were used for analysis and in the case of heat shock inducible transgenes were labeled by iEC (e.g., mCherry^iEC^, mCherry-dnTcf^iEC^).

### Heat shock and pharmacological treatments

In order to induce gene expression by heat shock, 26 hpf-old dechorionated embryos were incubated 40 min at 39 °C in pre-warmed E3 medium and analyzed at indicated developmental stages. For validation of the *Tg(hsp70l:loxP-STOP-loxP-mCherry-dntcf)*^*mu201*^ and *Tg(hsp70l:loxP-STOP-loxP-mCherry)*^*mu279*^ lines, embryos were incubated at 10 hpf and 15 hpf for 1 h at 39 °C and analyzed at 24 hpf.

For pharmacological treatments, dechorionated embryos were incubated from 5.5 to 24 hpf (for validation of S1pr antagonists), from 29 to 48 hpf or from 26 to 32 hpf in E3 medium supplemented with 150 µM Aphidicolin (A, Sigma-Aldrich)^33^, 100 µM Chloroquine (Sigma-Aldrich)^73,74^, 10 µM CYM5541 (Sigma-Aldrich), 10 µM CYM5542 (Tocris Bioscience), 80 µM Dynasore (Sigma-Aldrich)^74,75^, 20 mM Hydroxyurea (HU, Sigma-Aldrich)^33^, 20 µM IWR-1 (Sigma-Aldrich)^33^, 50 µM JTE013 (JTE, Cayman Chemical), 10 µM MG132 (Sigma-Aldrich)^74,76^, 100 µM NSC23766 (NSC, Sigma-Aldrich), 5 µg/mL Rho Activator II (Cytoskeleton)^59^, 2 µg/mL Rho inhibitor CT03 (Cytoskeleton)^59^, 100 µM TY52156 (TY, Tocris Bioscience) and 50 µM VPC23019 (VPC, Cayman Chemical). *Tg(hsp70l:loxP-STOP-loxP-mCherry-dntcf)*^*mu201*^ and *Tg(hsp70l:loxP-STOP-loxP-mCherry)*^*mu279*^ embryos were incubated directly after heat shock from 27 to 30 hpf with 12.5 µM and from 30 to 48 hpf with 25 µM VPC.

### Microinjections of fluorescent dye (angiography)

To analyze blood vessel perfusion, 2 nL 40 kDa FITC-Dextran 40 (1 mg/mL; TdB consultancy) were injected into the blood stream of anesthetized embryos at 48 hpf.

### Immunohistochemistry of zebrafish embryos

Immunohistochemistry was carried out as previously described^77^. Additionally, primary and secondary antibody dilutions were supplemented 1:1 with Pierce™ Immunostain Enhancer (Thermo Fisher Scientific). Antibodies used in this study: 1:400 rabbit anti-zf-VE-cadherin^77^, 1:1000 mouse anti–hZO-1 (Invitrogen, 339100^59^), 1:400 rabbit anti-Esama^23^, 1:200 rabbit anti-dnTcf (anti-TCF7L1; Proteintech Group, 14519-1-AP), 1:2000 goat anti-rabbit IgG Alexa Fluor 594 (Invitrogen, A-21207), 1:2000 goat anti-mouse IgG Alexa Fluor 647 (Invitrogen, A-21235), 1:2000 goat anti-mouse IgG Alexa Fluor 488 (Invitrogen, A-11001).

### Fluorescent in situ hybrisization (FISH)

Fluorescent in situ hybrisization (FISH) was adapted from refs. ^78,79^. Briefly: MeOH-fixed embryos were rehydrated, digested with Proteinase K (Roth, 7528.4, 10 µg/mL) for 12 min and refixed in 4% PFA. After washing with PBS containing 0.3% (v/v) Tween and pre-hybridization at 65 °C, embryos were incubated hybridization buffer containing digoxigenin (DIG)-labeled antisense probes at 65 °C for >12 h. Probe removal, blocking, and primary antibody incubation was performed according to the published protocol. Primary antibodies were POD-coupled anti-DIG (Roche, 1:400) and anti-GFP (Abcam, ab6556, 1:400), which was used for counter-staining GFP in transgenic embryos. After removal of the antibody solution, embryos were incubated for 60 min with TSA Plus Cy3 solution (Perkin Elmer, NEL744001KT), washed three times with PBS-Tween to remove excess staining solution and subjected to 1:500 goat anti-rabbit IgG Alexa Fluor 488 (Invitrogen) for 60 min. At last, embryos were washed with PBS-Tween >5 h and sequentially taken up in 75% Glycerol for confocal microscopy.

The antisense probes for *ve-cadherin*^80^ and *esama*^24,81^ were generated from plasmid templates using T3 or Sp6 polymerase and DIG-labelled UTP (2 h, 37 °C) and purified by precipitation^81^.

### Photoswitching and FACS isolation of brain capillary ECs

Photoswitching of *Tg(fli1:gal4)*^*ubs3*^*;(UAS:kaede)*^*rk8*^ embryos was performed using a Zeiss LSM 710 confocal microscope (Carl Zeiss, objective lenses: Plan-Apochromat ×20/0.8 M27) after embryo anesthesia with a low dose of tricaine. Embryos were mounted laterally in 1% low-melting agarose in glass-bottom Petri dishes (MatTek Corporation, Ashland, MA). Photoconversion of Kaede fluorescent protein was performed by scanning the selected region of interest with a 405 nm diode laser (100% laser, 5 iterations, 50 s). After photoconversion, embryos were manually removed from the agarose and placed in fresh egg water (0.3 g/L Instant Ocean Salt, 75 mg/L CaSO_4_; 1 mg/L Methylene Blue) additionally supplemented with N-Phenylthiourea (30 mg/L, Sigma-Aldrich) and covered with aluminum foil to protect from the light before trypsin treatment. After several washes in HBSS (Hank’s Balanced Salt Solution, Gibco) without Ca^2+^/Mg^2+^, zebrafish embryos dissociation was performed at 28.5 °C during 30 min using 2 mL of trypsin-LE select (Gibco). Dissociation was stopped by added 200 µL of FBS solution. After centrifugation, pellet was resuspended with HBSS with Ca^2+^/Mg^2+^ +5% FBS and filtered. Photoswitched ECs were isolated on FACS Aria II. Cells were immediately centrifuged, frozen by liquid nitrogen and stored at −80 °C.

### RNA sequencing of zebrafish brain capillary ECs

For RNA-seq, RNA was isolated from FACS sorted zebrafish CtAs ECs cells using the miRNeasy micro Kit (Qiagen) combined with on-column DNase digestion (DNase-Free DNase Set, Qiagen) to avoid contamination by genomic DNA. RNA and library preparation integrity were verified with a BioAnalyzer 2100 (Agilent) or LabChip Gx Touch 24 (Perkin Elmer). RNA amount was adjusted on number of isolated cells by FACS and ~250–500 pg total RNA was used as input for SMART-Seq® v4 Ultra® Low Input RNA Kit (Takara Clontech) for cDNA pre-amplification. Obtained full-length cDNA was checked on LabChip and fragmented by Ultrasonication by E220 machine (Covaris). Final Library Preparation was performed by Low Input Library Prep Kit v2 (Takara Clontech). Sequencing was performed on the NextSeq500 instrument (Illumina) using v2 chemistry, resulting in minimum of 30M reads per library with 1 × 75 bp single end setup. The resulting raw reads were assessed for quality, adapter content, and duplication rates with FastQC (available online at: http://www.bioinformatics.babraham.ac.uk/projects/fastqc). Reaper version 13–100 was employed to trim reads after a quality drop below a mean of Q20 in a window of 10 nucleotides^82^. Only reads between 30 and 150 nucleotides were cleared for further analyses. Trimmed and filtered reads were aligned versus the Zebrafish genome version DanRer10 (GRCz10.87) using STAR 2.4.0a with the parameter “—outFilterMismatchNoverLmax 0.1” to increase the maximum ratio of mismatches to mapped length to 10%^83^. The number of reads aligning to genes was counted with featureCounts 1.4.5-p1 tool from the Subread package^84^. Only reads mapping at least partially inside exons were admitted and aggregated per gene. Reads overlapping multiple genes or aligning to multiple regions were excluded. The Ensemble annotation was enriched with UniProt data (release 06.06.2014) based on Ensembl gene identifiers (activities at the Universal Protein Resource (UniProt)).

### RNA isolation from zebrafish embryos and RT-qPCR

RNA was isolated from zebrafish embryos with Trizol reagent (Thermo Fisher Scientific), and cDNA was generated using the SuperScript II reverse transcriptase kit (Invitrogen). Real-time quantitative PCR (RT-qPCR) was conducted using Power SYBR Green (Applied Biosystems) and the following primers (see also Supplementary Table 1): *axin2* fwd 5′-CCTGGAGGAGAGACTTCAAC-3′, *axin2* rev 5′-GAGCAAAGGCAGAGAATGGG-3′, *β-actin* fwd 5′-CTGGACTTCGAGCAGGAGAT-3′, *β-actin* rev 5′-GCAAGATTCCATACCCAGGA-3′, *β-catenin* fwd 5′-GCCGCCACCAAACAGGAG-3′, *β-catenin* rev 5′-CAGCAGCACACGTCACCACG-3′, *ccnd1* fwd 5′-CTGTGCGACAGACGTCAACT-3′, *ccnd1* rev 5′-GGTGAGGTTCTGGGATGAGA-3′, *lef1* fwd 5′-GTTGGACAGATGACCCCTCC-3′, *lef1* rev 5′-CTGTTTCACCTGTGGGTTGAC-3′.

### Mouse brain primary EC isolation and culture

For immunostaining or gene expression analysis of wild-type primary endothelial cells, C57BL/6J pups were used. To inactivate S1pr1 in the postnatal endothelium, *S1pr1*^lox/lox^^85^ and *Cdh5(PAC)CreERT2*^86^ mice were interbred. The resulting offspring were injected at P1 and P2 with 50 µg of 4-OHT and samples processed at P3.

Mouse brain primary microvascular fragment isolation and culture was performed as described^34^. In brief, brains were collected from mice at postnatal day 3 (P3) or adults (>8 weeks old) in ice-cold Dulbecco’s Modified Eagle’s Medium (DMEM, Life technologies 11965–092) supplemented with 1% penicillin/streptomycin (Gibco, 15140122) and L-Glutamine (Gibco, 25030081), hereafter termed dissection media. Brains were transferred to a Petri dish, where olfactory bulbs and cerebellum were removed. The hemispheres were mechanically disrupted to small pieces and further digested for 10 min at 37 °C in 0.5 mg/mL collagenase (Sigma-Aldrich, C6885) dissolved in dissection media (2 rounds of digestion for samples from adult animals). Next, the cell suspension was mixed with an equal volume of DMEM supplemented with 20% fetal bovine serum (FBS, Life Technologies), filtered through a 70 μm nylon cell strainer (Falcon, 352350) and centrifuged 5 min at 300 g. After centrifugation, the cell pellet was resuspended in dissection media supplemented with 0.5 mg/mL heparin (Sigma-Aldrich, H3149) and incubated for 40 min with CD31 (BD Pharmingen, 553370) -bound sheep anti-rat IgG magnetic Dynabeads (Life Technologies, 110.35) on a rotary wheel at room temperature. After incubation, microvascular fragments bound to magnetic beads were retrieved using a DynaMag-2 magnet (Life Technologies, 12321D), washed 5 times with heparin-supplemented dissection media and finally resuspended in TrypLE Select (Gibco, A12177-01) in order to dissociate them from the magnetic beads. After 12 min incubation at 37 °C, unbound beads were removed with the magnet and the suspension containing the microvascular fragments was mixed with EGM2-supplemented EBM2 media (Lonza, CC-3456 and CC-4176). After centrifugation (10 min, 300×*g*) the microvascular fragments were resuspended in EGM2–EBM2 media supplemented with puromycin (Sigma-Aldrich, P9620, 10 μg/mL) and plated on collagen-I-coated μ-Slide 8-well ibiTreat cell culture chambers (Ibidi, 80826) or 24-well cell culture plates. Hereafter, cells were kept in culture at 37 °C and 5% CO_2_. Twenty hours later, wells were washed three times with PBS to remove dead and unattached cells and fresh EGM2–EBM2 media supplemented with puromycin (5 μg/mL) was added. The next day, pharmacologic treatment started with drugs diluted in EGM2–EBM2 media.

IWR-1 (Sigma-Aldrich) and VPC23019 (VPC, Cayman Chemical) were dissolved in DMSO to a 10 mM concentration. Inhibition of Wnt signaling was achieved after 48 h treatment with 10 or 20 μM IWR-1 for endothelial cells from young pups or adult animals, respectively. Alternatively, 10 μM IWR-1-treated cells from young pups were exposed in parallel to 2 μM VPC to assess the effect of S1pr1 signaling blocking upon Wnt inhibition. Vehicle (DMSO) treated was used in all experiments as control.

### Immunohistochemistry of mouse brain primary EC

After inhibitor treatment, cells growing in 8-well ibiTreat cell culture chambers were fixed in 4% paraformaldehyde (PFA, Sigma-Aldrich, P6148) for 15 min at room temperature, washed three times with PBS and permeabilized with 0.2% Triton X-100 (Sigma-Aldrich, T8787) in PBS for 15 min. After washing three times with PBS, cells were blocked by incubation with 2% normal donkey serum (Abcam, ab7475) in PBS in a humidified chamber for 30 min. Primary antibodies (goat polyclonal anti-CD31, R&D Systems, AF3628, 1:100; rat monoclonal anti-VE-cadherin clone 11D4, BD Biosciences, 555289, 1:100; and rabbit polyclonal anti-ZO-1, Invitrogen, 402200, 1:100) were diluted in blocking buffer and incubated with the cells in a humidified chamber overnight at 4 °C. Wells were then washed three times with PBS and secondary antibodies (donkey anti goat Alexa Fluor 488, Invitrogen, A11055, 1:400; donkey anti rat Alexa Fluor Cy3, Jackson Immuno Research, 712-165-153, 1:400; and donkey anti rabbit Alexa Fluor 647, Invitrogen, A31573, 1:400) together with DAPI (Sigma-Aldrich, D9542, 1 μg/mL) were diluted in blocking buffer and incubated at room temperature for 2 h. After secondary antibody incubation, cells were washed as described above and covered with PBS until imaging.

### RT-qPCR of mouse brain primary ECs

After inhibitor treatment, cells growing in 24-well cell culture plates were collected in 350 μL RLT Plus Buffer (Qiagen, 1053393) supplemented with 1% *β*-mercaptoethanol (Sigma, M6250) and total RNA was isolated using the RNeasy Plus Micro Kit (Qiagen, 74034) following the manufacturer’s instructions. Whole RNA was assessed in terms of quantity and quality using Bioanalyzer 2000 (Agilent), reverse transcribed and converted to complementary DNA (cDNA) using the iScript cDNA synthesis kit (BioRad, 170-8890). The following FAM-conjugated TaqMan gene expression probes (all from Thermo Fisher Scientific) were used: *VE-cadherin* (*Cdh5*, Mm00486938_m1), *Axin2* (Mm01265780_m1), *Lef1* (Mm01310389_m1), and *Ccnd1* (Mm00432360_m1). VIC-conjugated *Actb* (4352341E) TaqMan probe was used to normalize gene expression. Quantitative PCR (qPCR) reactions were performed on a CFX96 Touch Real-Time PCR Detection System (BioRad) using the Sso Advanced Universal Probes Supermix (BioRad, 1725281). All relative gene expression analyses were performed using the 2^−ΔΔCt^ method in a minimum of four animals per group with triplicate reactions for each gene evaluated.

### Confocal microscopy

Confocal microscopy was performed using LSM780 and LSM880 microscopes (Carl Zeiss Microscopy GmbH; objective lenses: Plan-Apochromat ×20/0.8; LD C-Apochromat ×40/1.1 W Korr M27). For filopodia analysis in zebrafish at high resolution, images were acquired with the LSM880 Airyscan module. For life imaging of VE-cadherinTS embryos, the Online Fingerprinting mode of the LSM880 microscope was utilized. In general, PFA-fixed or living zebrafish embryos were embedded in 0.3% agarose, which was dissolved in E3 medium and additionally supplemented with N-Phenylthiourea (30 mg/L, Sigma-Aldrich) and Tricaine (19.2 mg/L, Sigma-Aldrich) for living embryos as previously described^59^. For time-lapse analysis, the agarose was additionally supplemented with IWR-1 or DMSO and a stable temperature of 28.5 °C was maintained using a heating chamber. Assembly of confocal stacks and time-lapse movies was conducted using Imaris 8/9 software (Bitplane). Quantification of signal intensity and volume was done using the Imaris surface-rendering algorithm.

### Statistical analysis

For all quantifications, statistical analysis was performed using Prism6 software (GraphPad). Graphs show mean ± standard deviation (SD) or standard error of the mean (SEM) and *p*-values were calculated using unpaired two-tailed Student’s *t*-test or one-way ANOVA for single and multiple comparisons as indicated for each experiment.

### Animal models

All animal experiments were performed in compliance with the relevant laws and institutional guidelines, were approved by local animal ethics committees and were conducted at the University of Münster and the MPI for Molecular Biomedicine with permissions granted by the Landesamt für Natur, Umwelt und Verbraucherschutz (LANUV) of North Rhine-Westphalia.

## Electronic supplementary material

Supplementary information

Description of Additional Supplementary Files

Supplementary Movie 1

Supplementary Movie 2

Supplementary Movie 3

Supplementary Movie 4

Supplementary Movie 5

Supplementary Movie 6

Supplementary Movie 7

Supplementary Movie 8

Supplementary Movie 9

Supplementary Movie 10

Supplementary Movie 11

Supplementary Movie 12

## Data Availability

The authors declare that the data supporting the findings of this study are available within the paper and its supplementary information files. The RNA sequencing data of this study have been deposited in NCBI’s Gene Expression Omnibus and are accessible through GEO Series accession number GSE121041. The rest of the data are available from the authors upon reasonable request.
