# Supplementary material for: Wnt/β-catenin signaling regulates VE-cadherin-mediated anastomosis of brain capillaries by counteracting S1pr1 signaling
Source: Nat Commun. 2018 Nov 19;9:4860. doi: 10.1038/s41467-018-07302-x (PMC6242933; doi:10.1038/s41467-018-07302-x)
Supplement: Supplementary file 1 — Supplementary information [file 41467_2018_7302_MOESM1_ESM.pdf]

Supplementary Information

Wnt/β-catenin signaling regulates VE-cadherin-mediated anastomosis of brain capillaries by counteracting S1pr1 signaling

(Hübner *et al.*)

Supplementary Figure 1

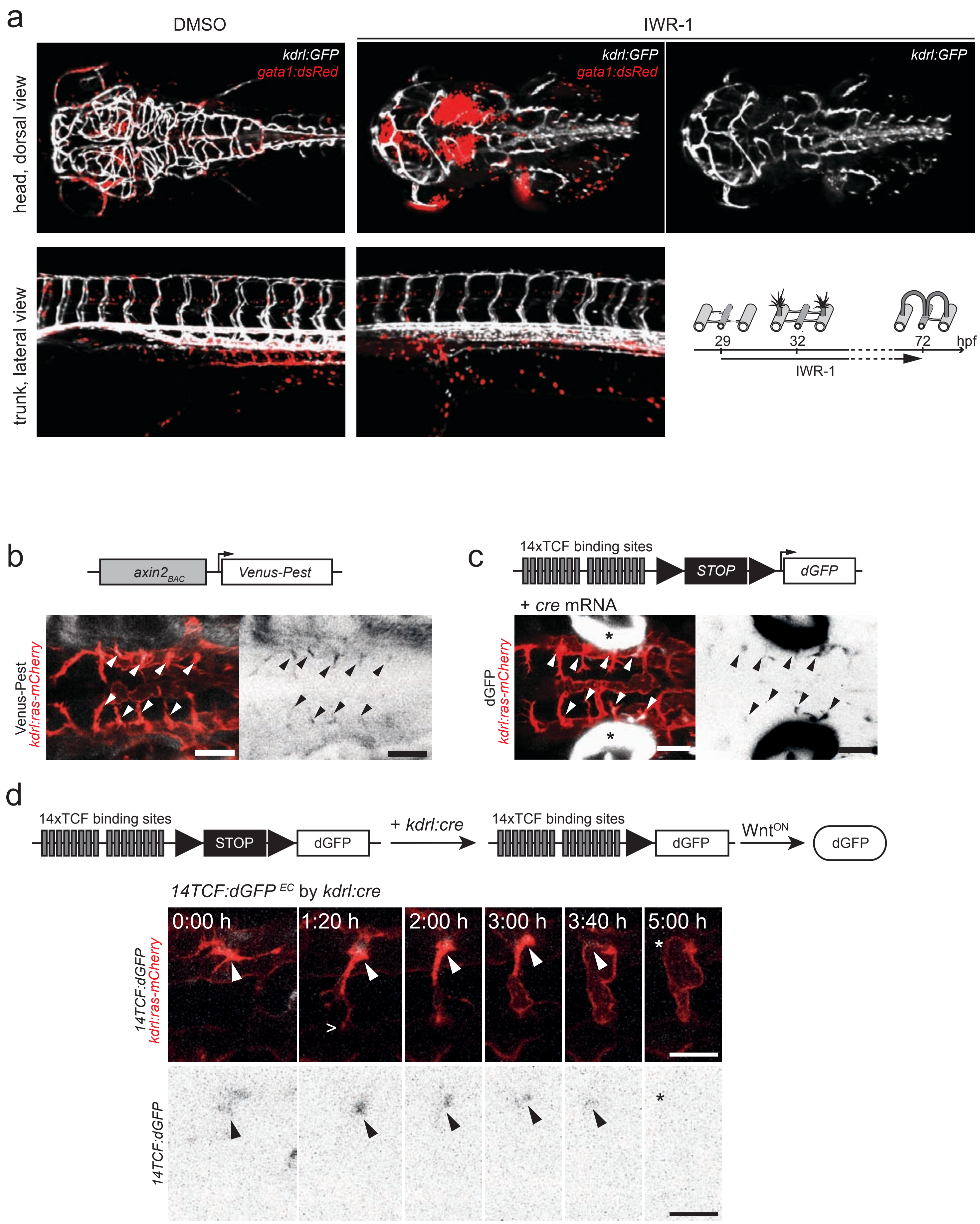

Supplementary Figure 1

Wnt signaling is active in brain ECs during angiogenesis

a) Wnt signaling inhibition by IWR-1 treatment caused severe vascular defects and hemorrhages in the brain, but not in the trunk of zebrafish embryos. Confocal images showing dorsal views of the head (anterior to the left) or lateral views of the trunk (anterior to the left) of 72 hpf-old embryos with ECs labeled with GFP (white, by *Tg(kdrl:GFP)*^{s843}) and erythrocytes labeled with dsRed (red, by *Tg(gata1:dsRed)*^{sd2}), which were treated with IWR-1 or DMSO from 29 to 72 hpf.

b,c) At 34 hpf, Wnt signaling reporters are expressed in all CtA sprouts in the hindbrain, indicating active Wnt signaling during sprout migration (arrowheads in b,c). Confocal images of embryos (dorsal views) co-expressing *Tg(kdrl:ras-mCherry)*^{s896} for labeling ECs (red) and β-catenin dependent Wnt signaling reporters expressing short-lived fluorophores: *Tg(axin2*$_{BAC}$*:Venus-Pest)*^{mu288} (b) and *Tg(14TCF:loxP-STOP-loxP-dGFP)*^{mu202} injected with *cre* mRNA (c). Asterisk in c marks Venus-Pest expression in the ear in *Tg(axin2*$_{BAC}$*:Venus-Pest)*^{mu28} embryos.

d) Vascular specific excision of the STOP cassette in *Tg(14TCF:loxP-STOP-loxP-dGFP)*^{mu202} embryos by *kdrl:cre*. Expression of dGFP indicates active Wnt signaling in ECs. Expression of dGFP was detected in CtA ECs starting during tip cell formation within the PHBC until lumen formation finished. Still images from a confocal time lapse movie of *Tg(14TCF:loxP-STOP-loxP-dGFP)*^{mu202}*; (kdrl:cre)*^{s898} double transgenic embryos with ECs labeled by *Tg(kdrl:ras-mCherry)*^{s896}. The dGFP channel is represented in inverted colors for better visualization. Arrows mark dGFP signal in CtA sprouts and open arrow indicates cell-cell-contact formation. Note, that the Wnt signaling responsive transgene (*14TCF:loxP-STOP-loxP-dGFP*) is heterozygous, which lead to weaker fluorescence signal compared to the homozygous transgene expression in Fig. 1b; CtA, central artery; ECs, endothelial cells; hpf, hours post fertilization; PHBC, primordial hindbrain channel.

Supplementary Figure 2

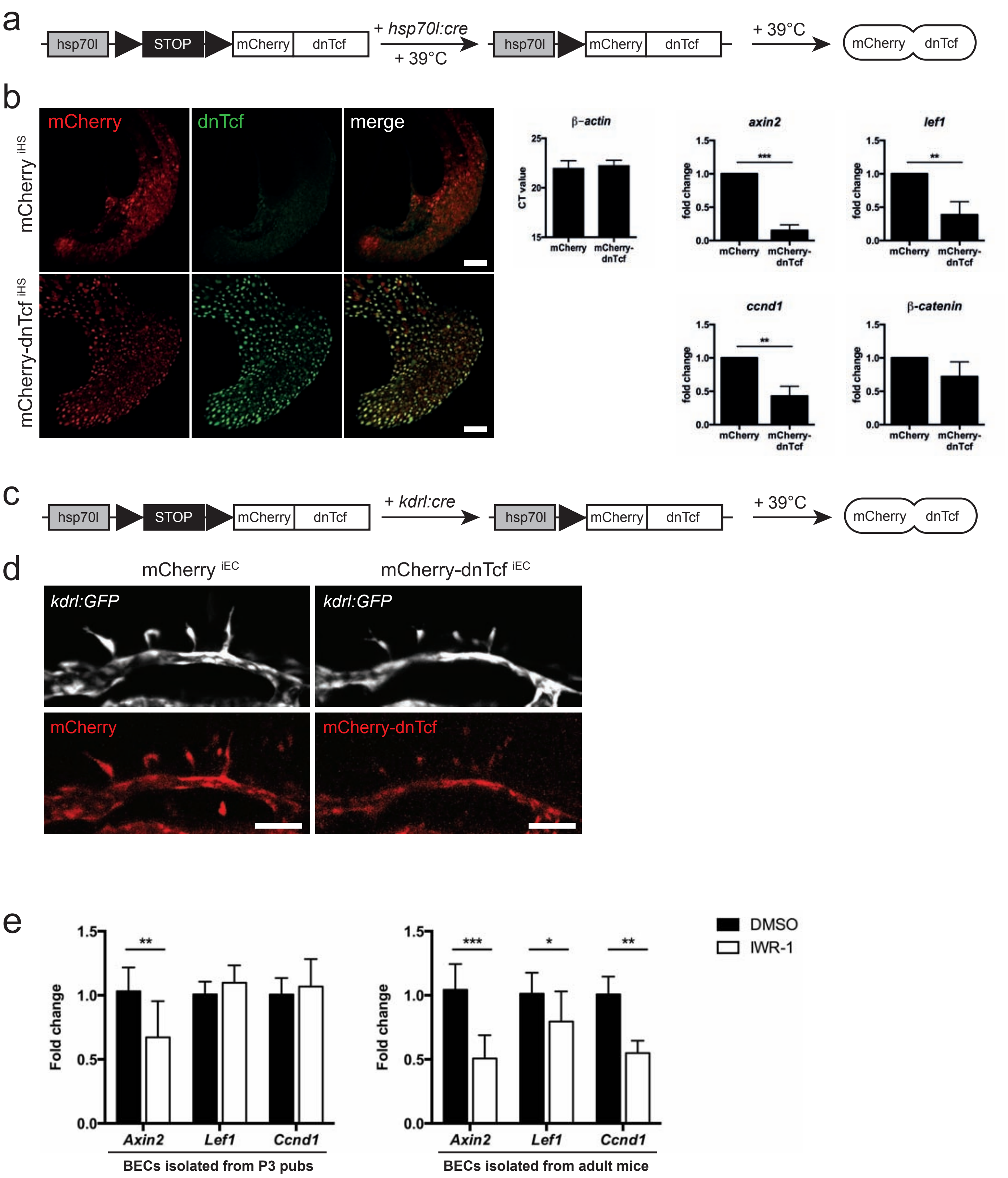

Supplementary Figure 2

Validation of transgenic Wnt-manipulating lines

a) For dnTcf expression in all cells, *Tg(hsp70l:loxP-STOP-loxP-mCherry-dntcf)^{mu201}* embryos were injected with *cre* mRNA and subjected to heat shock to induce mCherry-dnTcf expression (referred as mCherry-dnTcf^{iHS}).

b) Overexpression of dnTcf robustly blocks expression of Wnt target genes. Induction of mCherry-dnTcf in all cells by heat shock can be detected by immunostaining in 24 hpf-old *Tg(hsp70l:loxP-STOP-loxP-mCherry-dntcf)^{mu201}* but not in *Tg(hsp70l:loxP-STOP-loxP-mCherry)^{mu279}* embryos. Wnt signaling target genes *axin2*, *lef1* and *ccnd1* were down regulated after mCherry-dnTcf expression, but the expression of *β-catenin* was not significantly affected. Values represent mean ± SD. *p<0.05, **p<0.01, ***p<0.001, Student's t-test of three independent experiments.

c) For EC-specific expression of dnTcf, *Tg(hsp70l:loxP-STOP-loxP-mCherry-dntcf)^{mu201}* embryos were mated to *Tg(kdrl:cre)^{s898}* and mCherry-dnTcf expression was induced by heat shock at 26 hpf (referred as mCherry-dnTcf^{iEC}).

d) Expression of mCherry^{iEC} or mCherry-dnTcf^{iEC} co-localized with GFP expression from *Tg(kdrl:GFP)^{s843}* in CtAs and PHBC of 32 hpf-old embryos. Therefore, the heat shock protocol is suitable to induce mCherry-dnTcf during CtA angiogenesis.

e) Analysis of expression levels of Wnt target genes in DMSO or IWR-1 treated mouse primary BECs isolated from P3 or adult animals by RT-qPCR (P3: *n*=4, adult: *n*=7). Values in represent mean ±SD, *p<0.05, **p<0.01, ***p<0.001, *n*, number of biological replicates.

Confocal images of zebrafish embryos show lateral views (anterior to the left). BECs, brain ECs, CtA, central artery; ECs, endothelial cells; PHBC, primordial hindbrain channel; Scale bars: 50 μm.

Supplementary Figure 3

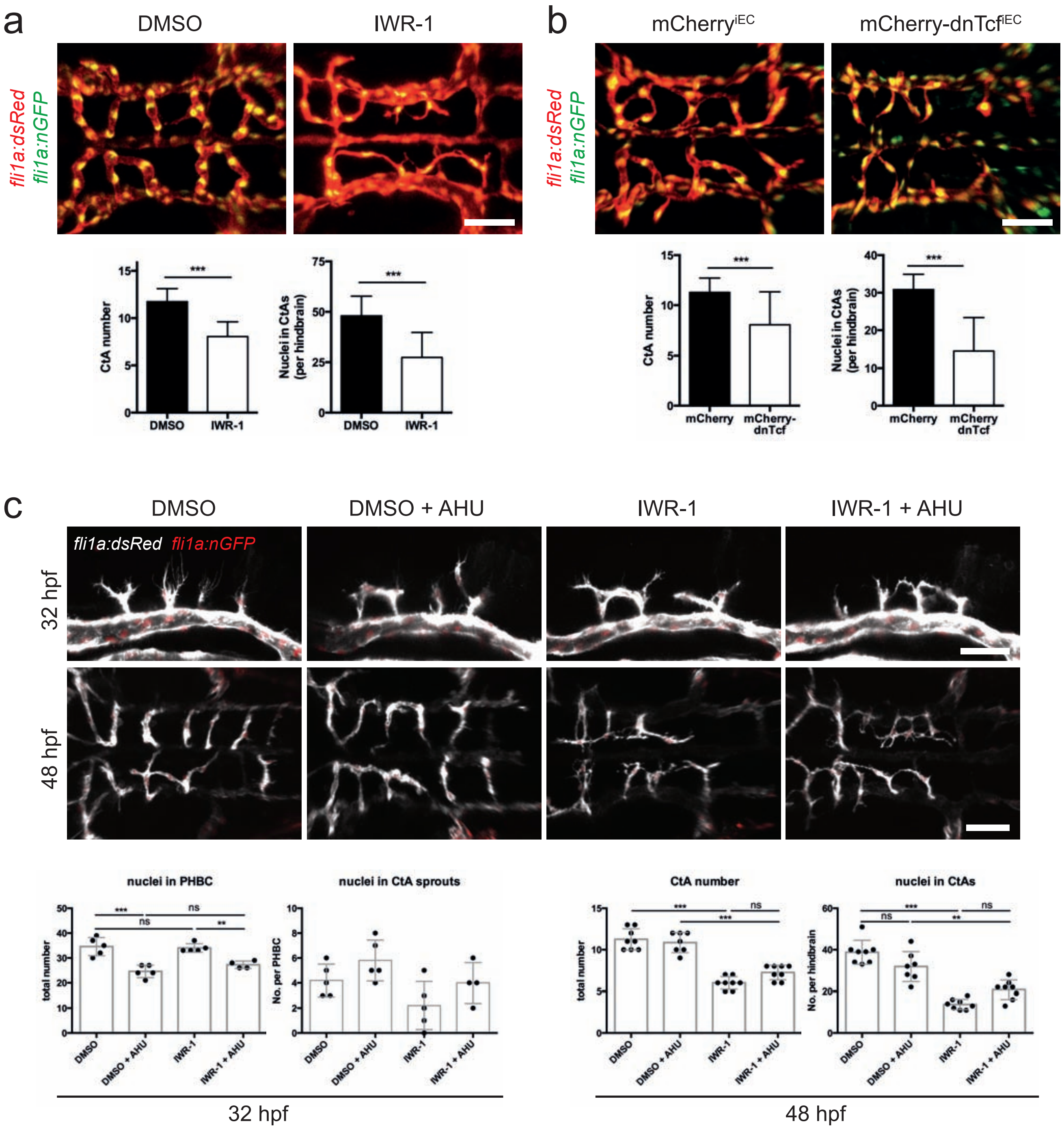

Supplementary Figure 3

Wnt signaling impacts EC number independent of proliferation

a,b) Inhibition of Wnt signaling by IWR-1 treatment (a) or by EC-specific dnTcf expression induced by heat shock at 26 hpf (b) affected EC numbers in CtAs.

a) IWR-1 treatment from 29 to 48 hpf resulted in decreased total number of CtAs and also reduced EC number within CtAs compared to control (DMSO: CtA number n=35, EC number n=39; IWR-1: CtA number n=20 EC number n=22).

b) Expression of mCherry-dnTcf^{iEC} resulted in decreased total number of CtAs and reduced EC number within CtAs compared to mCherry^{iEC} (mCherry^{iEC}: CtA number n=39, EC number n=40; mCherry-dnTcf^{iEC}: CtA number n=12, EC number n=14).

c) Decreased EC numbers after Wnt signaling inhibition did not result from decreased EC proliferation. Embryos were treated with DMSO (control), IWR-1, AHU or IWR-1+AHU from 26 to 32 hpf or from 29 to 48 hpf. Inhibition of proliferation by AHU decreased the number of ECs in the PHBC at 32 hpf (n=5), but had no effect on the EC number within CtAs at 48 hpf, which was only affected by IWR-1 treatment (n=5). Confocal images show dorsal views of 48 hpf-old embryos and lateral views of 32 hpf-old embryos (anterior to the left) with GFP expression from *Tg(fli1a:nGFP)*^{y7} and dsRed expression from *Tg(fli1a:dsRed)*^{um13}. Values represent mean ±SD. *p<0.05, **p<0.01, ***p<0.001, Student's t-test (a,b) or One-way ANOVA (c); n, number of analyzed embryos; CtAs, central arteries; ECs, endothelial cells; PHBC, primordial hindbrain channel; Scale bars: 50 µm.

Supplementary Figure 4

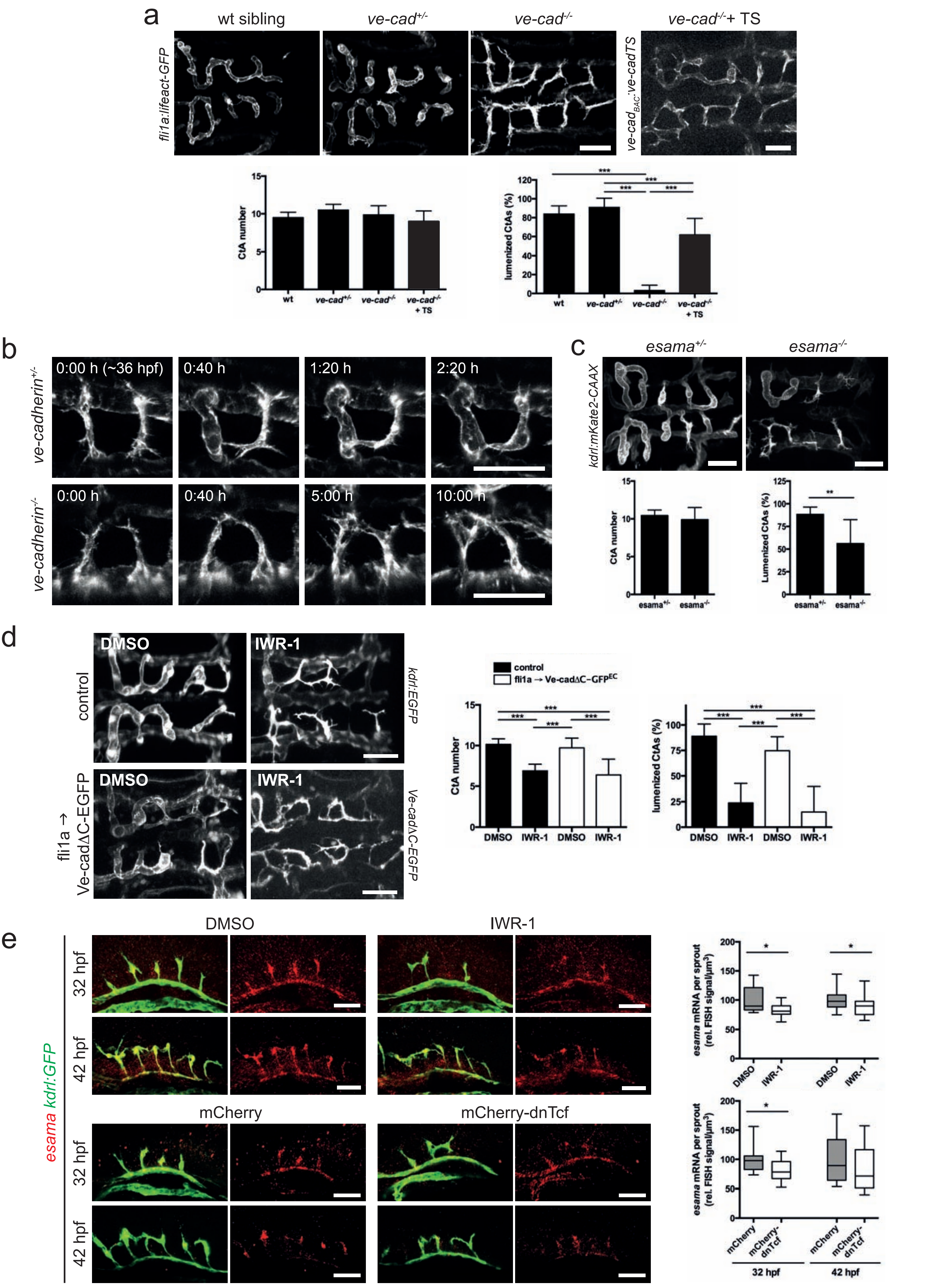

Supplementary Figure 4

VE-cadherin and Esama are required for CtA anastomosis

a) Lumen formation was completely impaired in homozygous *ve-cadherin*^{ubs8} mutant embryos at 48 hpf, while the number of CtAs remained unaffected. Expression of an intracellular modified VE-cadherinTS in *ve-cadherin*$^{ubs8/ubs8}$ mutants partially restored the lumen formation defects (wt: *n*= 2; *ve-cadherin*$^{ubs8/+}$: *n*=8; *ve-cadherin*$^{ubs8/ubs8}$: *n*=7; *ve-cadherin*$^{ubs8/ubs8}$ + TS: *n*=22).

b) Still images from time-lapse movies of *ve-cadherin*^{ubs8} mutant embryos expressing *Tg(fli1a:lifeact-GFP)*^{mu240}. In homozygous *ve-cadherin*^{ubs8} mutants anastomosis of CtAs was completely abolished. Heterozygous siblings did not exhibit anastomosis defects (*ve-cadherin*$^{ubs8/ubs8}$: *n* = 4; siblings: *n* = 4).

c) Homozygous *esama*^{ubs19} mutants showed decreased proportion of lumenized CtAs, but no changes in CtA numbers (*esama*$^{+/ubs19}$: *n*=9; *esama*$^{ubs19/ubs19}$: *n*=10).

Confocal images show dorsal views (anterior to the left) and GFP expression of *Tg(fli1a:lifeact-GFP)*^{mu240} (a,b) or mKate expression of *Tg(kdrl:mKate-CAAX)*^{ubs16} (c).

d) Overexpression of a truncated VE-cadherin version (VE-cadΔC-EGFP) did not restore lumen formation defects in Wnt-depleted embryos. *Tg(kdrl:GFP)*^{s843} (control) or *Tg(fli1a:Gal4)*^{ubs3}*;(UAS: VE-cadΔC-EGFP)*^{ubs12} embryos were treated from 29 to 48 hpf with IWR-1 or DMSO (control: DMSO: *n*=7, IWR-1: *n*=8; VE-cadΔC: DMSO: *n*=14, IWR-1: *n*=13). Values in a-d represent mean ± SD. *p<0.05, **p<0.01, ***p<0.001, Student's t-test; *n*, number of analyzed embryos.

e) Inhibition of Wnt signaling by incubation with IWR-1 or heat shock induced EC-specific dnTcf expression did not severely affect *esama* mRNA expression at 32 or 42 hpf. Expression of *esama* in CtA sprouts was slightly decreased after IWR-1 treatment or mCherry-dnTcf^{iEC} expression (32hpf: DMSO: *n*=15, *N*=5 and IWR-1: *n*=9, *N*=5, mCherry^{iEC}: n=16, N=6 and mCherry-dnTcf^{iEC}: n=10, N=5; 42hpf: DMSO: n=24, N=5 and IWR-1: *n*=16, *N*=5, mCherry: *n*=16, *N*=5 and mCherry-dnTcf: *n*=9, *N*=4). Confocal images show lateral views (anterior to the left) of *Tg(kdrl:GFP)*^{s843} embryos following display whole mount fluorescent *in situ* hybridization in combination with anti-GFP immunostaining. Quantifications were represented by Box-and-Whisker plots with median (center line), 25th and 75th percentiles (bounds of box) and Min-to-Max (whiskers), *p<0.05, **p<0.01, ***p<0.001, Student's t-test; *n*, number of CtA volumes analyzed; *N*, number of embryos analyzed; CtAs, central arteries; ECs, endothelial cells; PHBC, primordial hindbrain channel, TS, tension sensor; Scale bars: 50 µm.

Supplementary Figure 5

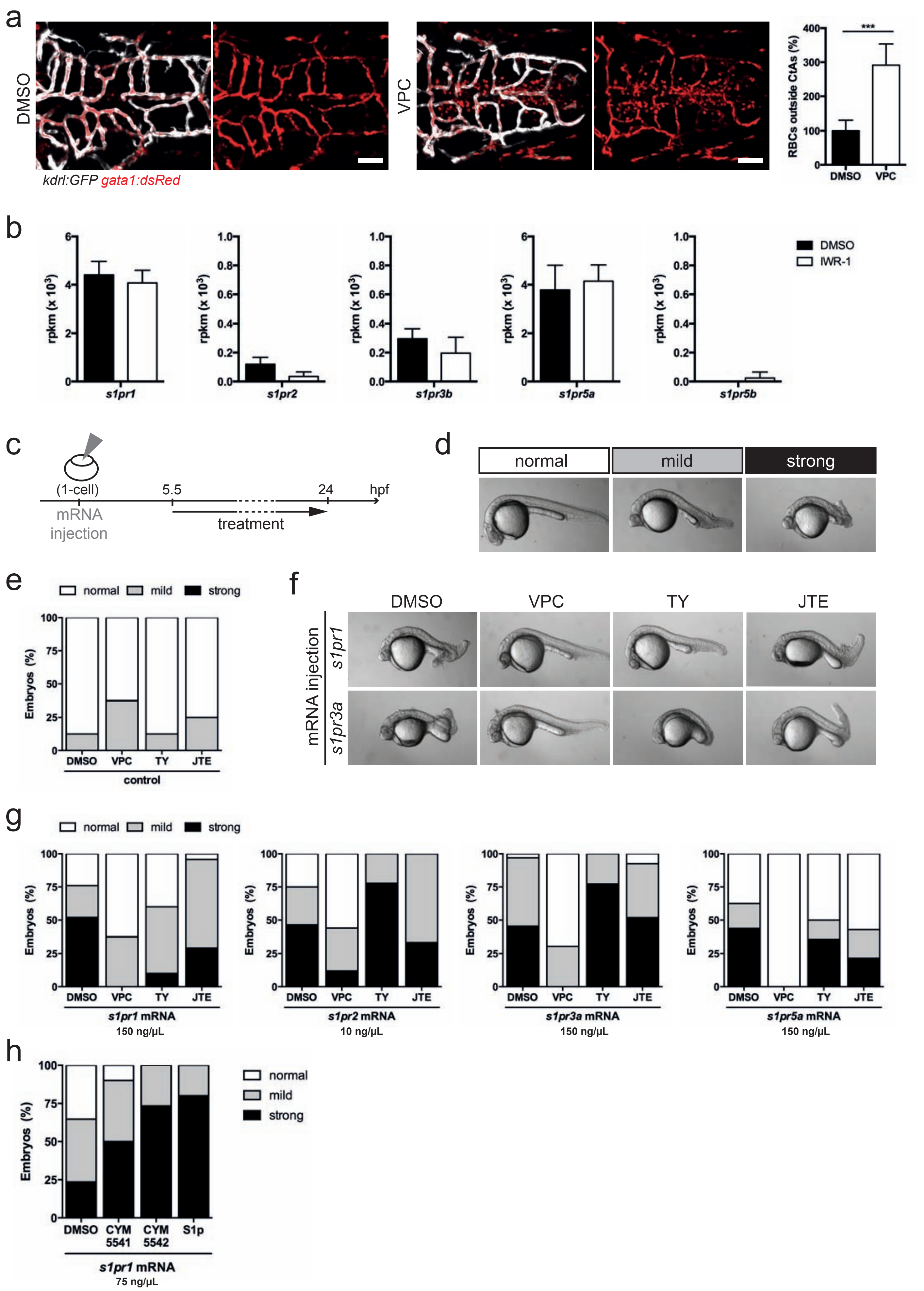

Supplementary Figure 5

Pharmacological targeting of S1pr receptors in zebrafish

a) Pharmacological inhibition of S1pr signaling causes extravasation of erythrocytes (red blood cells, RBCs). Embryos expressing GFP in ECs (by *Tg(kdrl:GFP)*^{s843}) and dsRed in RBCs (by *Tg(gata1:dsRed)*^{sd2}) were treated with VPC23019 (VPC) or DMSO from 48 to 72 hpf (DMSO: *n*=9; VPC: *n*=9).

b) Expression levels of *s1pr's* expressed in CtA ECs were not significantly changed by inhibition of Wnt signaling (RNA sequencing, see methods).

c-g) S1pr's (S1pr1, S1pr2, S1pr3a and S1pr5a) were overexpressed by mRNA injection into single-cell stage embryos. Rescue experiments were performed by treatment with VPC23019 (VPC, described S1pr1 and S1pr3 antagonist), TY52156 (TY, described S1pr3 antagonist) and JTE013 (JTE, described S1pr2 antagonist) from 5.5 hpf to 24 hpf (c,f,g). Embryos were sorted in categories according to phenotype (d): "normal" (white): wild type appearance, "mild": little developmental delay, minor anterior-posterior body axis formation defects (>60% of head and tail formed, intact eye and capacity of embryonic reflex),"strong": severe developmental delays, profound anterior-posterior axis defects (<60% of head and tail formed, obvious deformation or complete lack of tissues and organs, such as brain, eye and tail). VPC (described as S1pr1/S1pr3 antagonist) rescued phenotypic defects of all *s1pr* overexpression scenarios (f,g), TY (described as S1pr3 antagonist) specifically rescued *s1pr1* overexpression in zebrafish. The described S1pr2 antagonist JTE, did not restore phenotypic defects in the tested overexpression scenarios (e: control: DMSO: *n*=8, VPC: *n*=8, TY: *n*=8, JTE: *n*=8; f,g: *s1pr1* mRNA: DMSO: *n*=25, VPC: *n*=16, TY: *n*=20, JTE: *n*=24; *s1pr2* mRNA: DMSO: *n*=28, VPC: *n*=25, TY: *n*=27, JTE: *n*=30; *s1pr3a* mRNA: DMSO: *n*=33, VPC: *n*=33, TY: *n*=22, JTE: *n*=27; *s1pr5a* mRNA: DMSO: *n*=16, VPC: *n*=8, TY: *n*=14, JTE: *n*=14).

h) After mRNA injection overexpressed S1pr1 can be stimulated by S1p co-injection or treatment with pharmacological agonists. S1pr3 agonist CYM5541 or S1pr1 agonist CYM5542 aggravate phenotypic defects in comparison to control (*s1pr1* mRNA: DMSO: *n*=17, CYM5541: *n*=20, CYM5542: *n*=15, S1p: *n*=30). Brightfield images (d,f) show lateral views (anterior to the left) of representative zebrafish embryos at 24 hpf.

Values represent mean ±SD. *p<0.05, **p<0.01, ***p<0.001, student's t-test; *n*, number of analyzed embryos.

Supplementary Figure 6

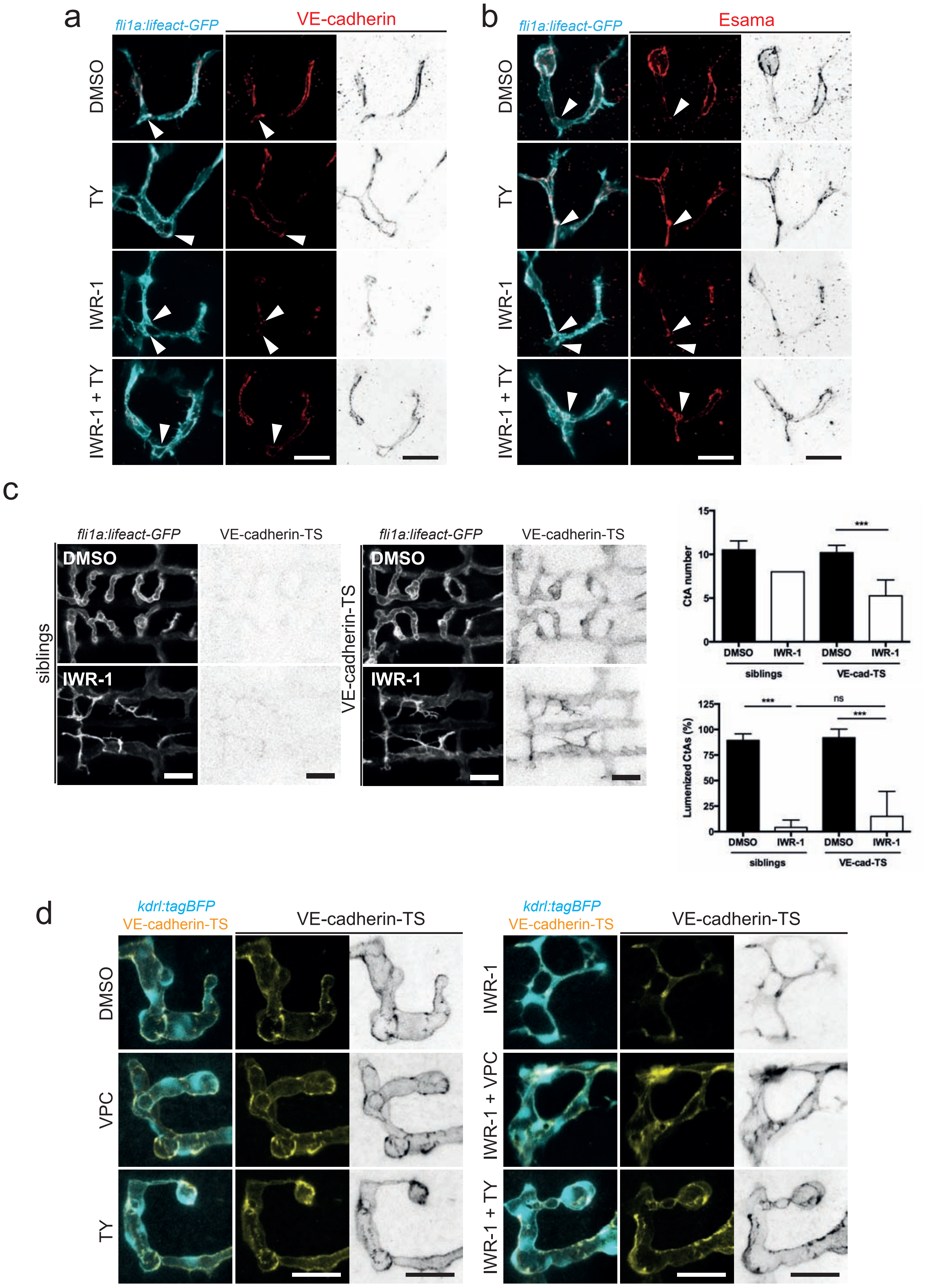

Supplementary Figure 6

Wnt and S1pr signaling regulate VE-cadherin localization

a,b) Inhibition of S1pr1 signaling together with blocking Wnt signaling rescued VE-cadherin (a) and Esama (b) protein levels at the cell-cell junctions. Immunostaining was performed for VE-cadherin (a, red) and Esama (b, red) of 42 hpf-old embryos after single or co-treatment with IWR-1 and TY. Junction localization of VE-cadherin and Esama (arrows show anastomosis rings) was reduced after IWR-1 treatment, not affected by TY treatment and restored in co-treated embryos.

c) Expression of the VE-cad-TS in wild type embryos does not restore or worsen the lumen formation defects caused by Wnt signaling inhibition. *Tg(ve-cad*$_{BAC}$*:ve-cadTS)*^{uq11bh} embryos (and control siblings) were treated with DMSO or IWR-1 from 29 to 48 hpf (sibling: DMSO: *n*=6, IWR-1: *n*=3; VE-cad-TS: DMSO: *n*=5, IWR-1: *n*=8). Values represent mean ±SD. *p<0.05, **p<0.01, ***p<0.001, One-way ANOVA; *n*, number of analyzed embryos.

d) Blocking of S1pr signaling rescued localization of VE-cad-TS in cell-cell junctions as well as lumen formation in Wnt-depleted embryos. Life imaging of VE-cad-TS embryos (*Tg(ve-cad*$_{BAC}$*:ve-cadTS)*^{uq11bh}) at 48 hpf following single or co-treatment with IWR-1 and S1pr antagonist VPC or S1pr1 antagonist TY starting from 29 hpf.

Confocal images show dorsal views (anterior to the left) and ECs labeled by *Tg(fli1a:lifeact-GFP)*^{mu240} (a-c) or *Tg(kdrl:tagBFP)*^{mu293} (d). CtAs, central arteries; S1pr, Sphingosine-1-phosphate receptor, TS, tension sensor, VE-cad, VE-cadherin; Scale bars: (a,b,d) 30 µm, (c) 50 µm.

Supplementary Figure 7

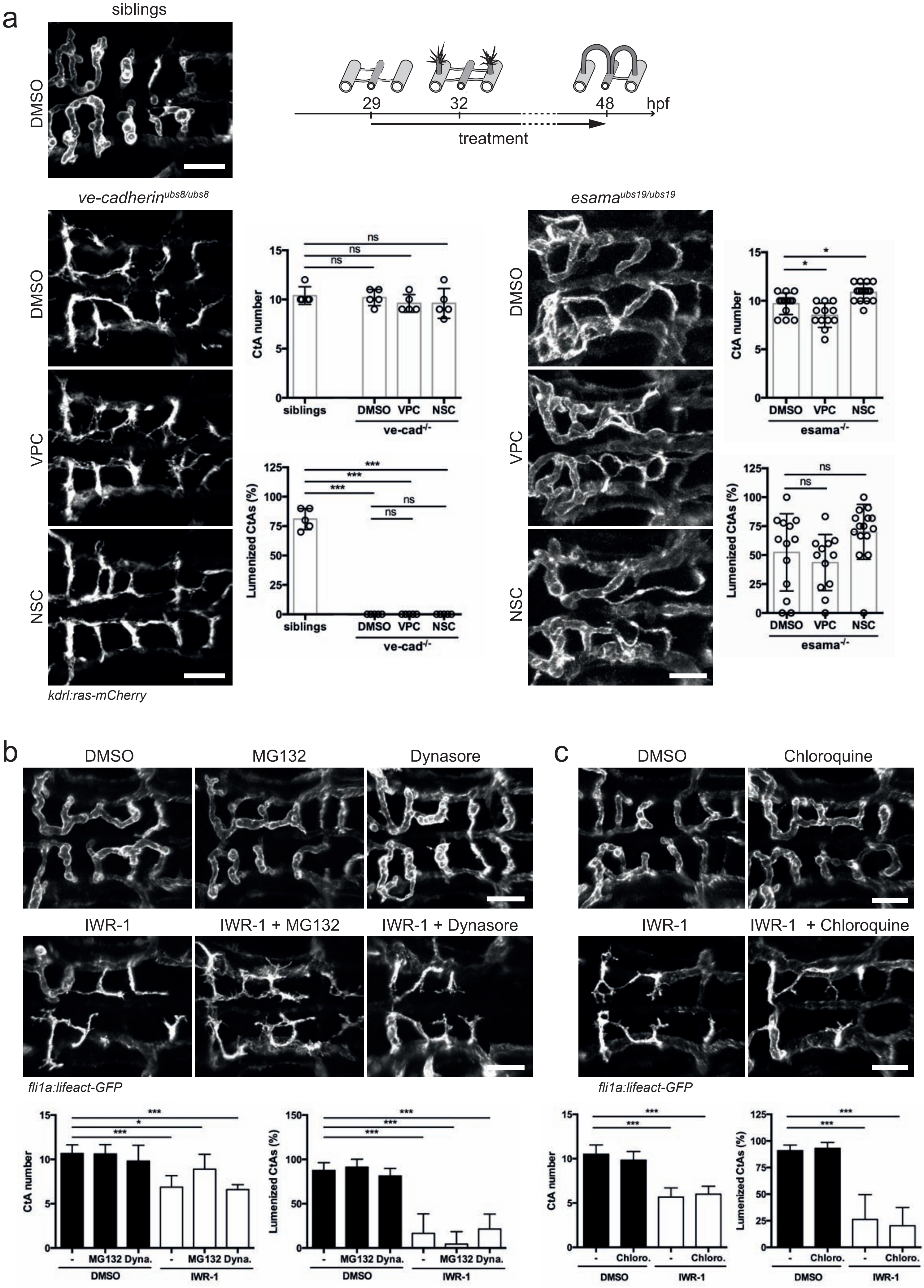

Supplementary Figure 7

Wnt and S1pr1 signaling act upstream of VE-cadherin function

a) Pharmacologic inhibition of S1pr signaling or Rac1 activity does not rescue CtA formation defects in *ve-cadherin* or *esama* mutant embryos. Treatment of *ve-cadherin* or *esama* mutant embryos with DMSO, VPC (S1pr antagonist) or NSC (Rac1 inhibitor) from 29 to 48 hpf (siblings: *n*=5; *ve-cadherin*$^{-/-}$: DMSO: *n*=5, VPC: *n*=5, NSC: *n*=5; *esama*$^{-/-}$: DMSO: *n*=13, VPC: *n*=15, NSC: *n*=12).

b,c) Embryos were single or co-treated with IWR-1, MG132 (proteasome inhibitor,b), Dynasore (dynamin inhibitor, b) or Chloroquine (lysosome inhibitor, c) from 29 to 48 hpf. Treatment with IWR-1 reduced CtA number and proportion of lumenized CtAs compared to control. Co-treatment with MG132, Dynasore or Chloroquine could not restore lumen formation defects caused by IWR-1 (b: DMSO: *n*=10; MG132: *n*=10; Dynasore: *n*=5; IWR-1: *n*=10; IWR-1 + MG132: *n*=10; IWR-1 + Dynasore: *n*=5; c: DMSO: *n*=6; Chloroquine: *n*=6; IWR-1: *n*=6; IWR-1 + Chloroquine: *n*=6).

Confocal images show dorsal views (anterior to the left) and ECs labeled by *Tg(kdrl:ras-Cherry)*^{s896} (a). or *Tg(fli1a:lifeact-GFP)*^{mu240} (b,c). Values represent mean ± SD. *p<0.05, **p<0.01, ***p<0.001, One-way ANOVA; *n*, number of analyzed embryos; CtAs, central arteries; S1pr, Sphingosine-1-phosphate receptor; Scale bars: 50 µm.

Supplementary Figure 8

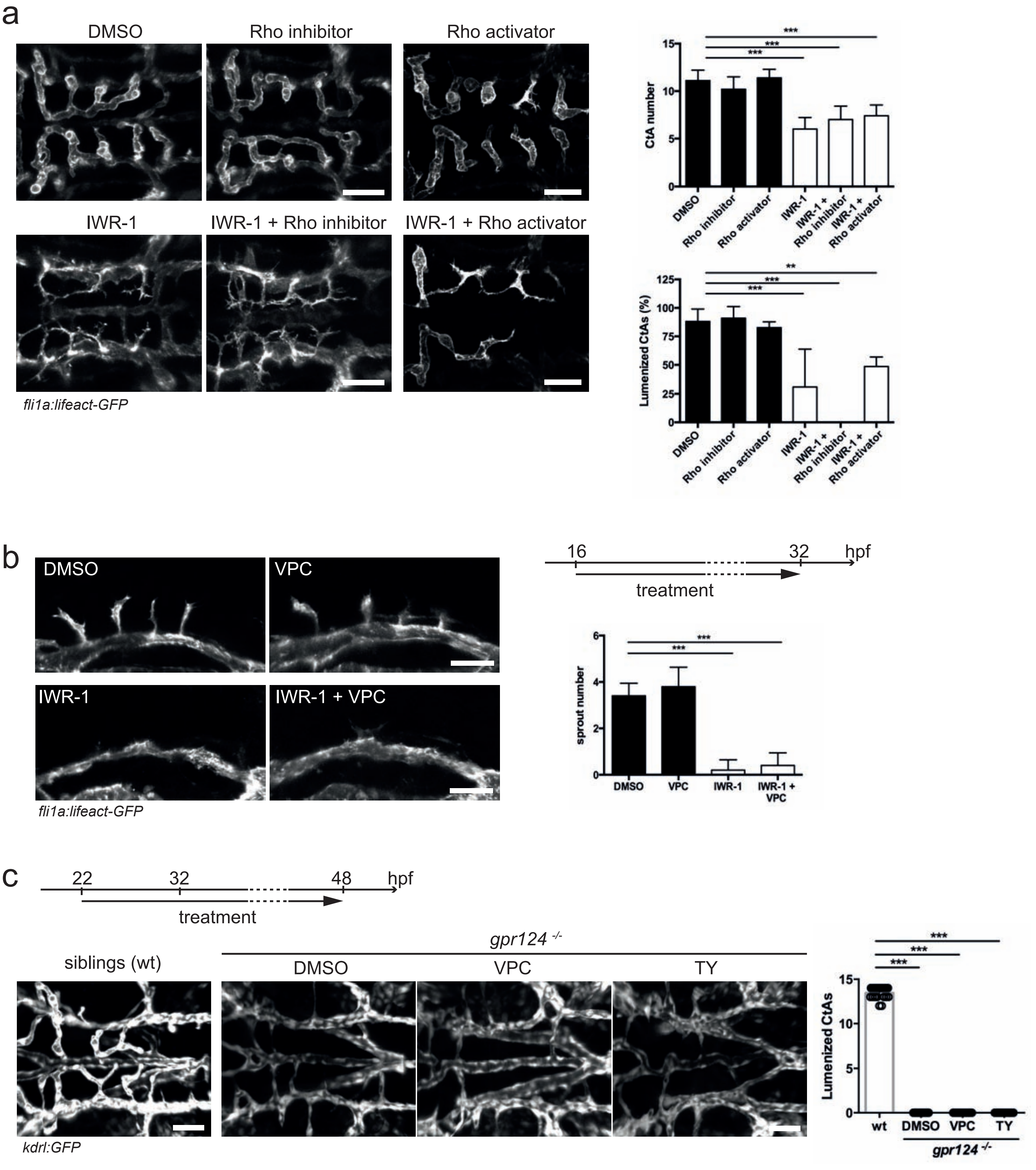

Supplementary Figure 8

Manipulation of Rho does not rescue Wnt-depleted embryos

a) Embryos were single or co-treated with IWR-1, Rho inhibitor or Rho activator from 29 to 48 hpf. CtA number and proportion of lumenized CtAs were reduced after treatment with IWR-1 and could not be rescued by co-treatment with Rho activator or Rho inhibitor (DMSO: *n*=10; Rho inhibitor: *n*=5; Rho activator: *n*=5; IWR-1: *n*=9; IWR-1 + Rho inhibitor: *n*=4; IWR-1 + Rho activator: *n*=5).

b) Pharmacologic inhibition of S1pr signaling does not rescue CtA sprouting defects in Wnt-depleted embryos. *Tg(fli1a:lifeact-GFP)*^{mu240} embryos were single or co-treated with IWR-1 and VPC starting from 16 ss (17 hpf) to 32 hpf. CtA tip cell formation was abolished in IWR-1 and IWR-1+VPC co-treated embryos, but not affected in VPC single-treated embryos in comparison to control (DMSO: *n*=5; VPC: *n*=5; IWR-1: *n*=5; IWR-1 + VPC: *n*=5).

c) Pharmacologic inhibition of S1pr signaling does not rescue CtA formation defects in *gpr124* mutant embryos. Treatment of gpr124 mutant embryos with DMSO, VPC or TY from 22 to 48 hpf (wt: *n*=10; *gpr124*$^{-/-}$: DMSO: *n*=10, VPC: *n*=8, TY: *n*=8).

Confocal images show dorsal views (a,c) or lateral views (b) (anterior to the left) and GFP expression from *Tg(fli1a:lifeact-GFP)*^{mu240}(a,c) or *Tg(kdrl:GFP)*^{s843}(b). Values represent mean ± SD. *p<0.05, **p<0.01, ***p<0.001, one-way ANOVA; *n*, number of analyzed embryos; CtAs, central arteries; S1pr, Sphingosine-1-phosphate receptor; Scale bars: 50 µm.

Supplementary Table 1

**Primer**	**Sequence (5' –> 3')**
s1pr1 fwd	ATGGATGACCTAATCGCC
s1pr1 rev	CGAGACGAAAAAGTTCACG
s1pr2 fwd	ATGACTACTTGCCGTCTG
s1pr2 rev	GGGATCTGCAAACACTTGG
s1pr3a fwd	ATGGATGACGAGCTTGAACC
s1pr3a rev	TCAGAACTTCCCCAAAGCG
s1pr5a fwd	GGTCAGCAGAAGTGAAATGG
s1pr5a rev	CAGACTTGTTTACTTGGCAG
s1pr1 CRISPR genotyping fwd	TCTATACTGCCAACATCCTG
s1pr1 CRISPR genotyping rev	CTGATGAGCATGAAGACCC
qPCR *axin2* fwd	CCTGGAGGAGAGACTTCAAC
qPCR *axin2* rev	GAGCAAAGGCAGAGAATGGG
qPCR *β-actin* fwd	CTGGACTTCGAGCAGGAGAT
qPCR *β-actin* rev	GCAAGATTCCATACCCAGGA
qPCR *β-catenin* fwd	GCCGCCACCAAACAGGAG
qPCR *β-catenin* rev	CAGCAGCACACGTCACCACG
qPCR *ccnd1* fwd	CTGTGCGACAGACGTCAACT
qPCR *ccnd1* rev	GGTGAGGTTCTGGGATGAGA
qPCR *lef1* fwd	GTTGGACAGATGACCCCTCC
qPCR *lef1* rev	CTGTTTCACCTGTGGGTTGAC
