# Supplementary material for: Wnt/β-catenin signaling regulates VE-cadherin-mediated anastomosis of brain capillaries by counteracting S1pr1 signaling
Source: Nat Commun. 2018 Nov 19;9:4860. doi: 10.1038/s41467-018-07302-x (PMC6242933; doi:10.1038/s41467-018-07302-x)
Supplement: Supplementary file 2 — Description of Additional Supplementary Files [file 41467_2018_7302_MOESM2_ESM.docx]

**Description of Additional Supplementary Files**

File name: Supplementary Movie 1

**Description: Wnt signaling reporter is continuously active during brain capillary angiogenesis**

Confocal time-lapse movie of a representative *Tg(14TCF:loxP-STOP-loxP-dGFP)^mu202^* embryo, which expressed Cre recombinase in all cells, with ECs labeled by *Tg(kdrl:ras-mCherry)^s896^* starting at around 28 hpf. Expression of dGFP marks active Wnt signaling and can be detected within CtA ECs continuously during CtA angiogenesis (see Fig 1a) until lumen formation occurred. Still images and inverted colors were displayed in Fig. 1b. Confocal stacks were acquired in 30 min intervals. Dorsal view, with anterior to the left. CtAs, central arteries; hpf, hours post fertilization.

File name: Supplementary Movie 2

**Description: Wnt signaling reporter is active in brain capillary endothelial cells**

Confocal time-lapse movie of a representative *Tg(14TCF:loxP-STOP-loxP-dGFP)^mu202^;(kdrl:cre)^s898^* embryo, expressing Cre recombinase in ECs, starting at around 28 hpf with ECs labeled by *Tg(kdrl:ras-mCherry)^s896^*. Expression of dGFP marks active Wnt signaling and can be detected exclusively in CtA ECs during CtA angiogenesis. Confocal stacks were acquired in 20 min intervals. Still images and inverted colors were displayed in Supplementary Fig. 1d. Dorsal view, with anterior to the left. CtAs, central arteries; ECs, endothelial cells; hpf, hours post fertilization.

File name: Supplementary Movie 3

**Description: Brain capillary spouting in control embryos**

Confocal time-lapse movie of a representative wild type embryo (DMSO treatment at 26 hpf) with GFP expression from *Tg(fli1a:lifeact-GFP)^mu240^* starting at around 32 hpf shows normal CtA sprouting and sprout invasion into the brain parenchyma. Confocal stacks were acquired in 15 min intervals. Lateral view, with anterior to the left. CtAs, central arteries; hpf, hours post fertilization.

File name: Supplementary Movie 4

**Description: Brain capillary spouting is not affected in Wnt-depleted embryos**

Confocal time-lapse movie of a representative Wnt-depleted embryo (IWR-1 treatment at 26 hpf) with GFP expression from *Tg(fli1a:lifeact-GFP)^mu240^* starting at around 32 hpf shows normal CtA sprouting and sprout invasion into the brain parenchyma. Confocal stacks were acquired in 15 min intervals. Lateral view, with anterior to the left. CtAs, central arteries; hpf, hours post fertilization.

File name: Supplementary Movie 5

**Description: Brain capillary angiogenesis in control embryos (DMSO treated)**

Confocal time-lapse movie of a representative wild type embryo (DMSO treatment at 26 hpf) with GFP expression from *Tg(fli1a:lifeact-GFP)^mu240^* starting at around 32 hpf shows normal sprouting, invasion and anastomosis of CtA sprouts. Lumen formation occurred in average in less than 2 hours after cell-cell contact formation. Confocal stacks were acquired in 15 min intervals. Dorsal view, with anterior to the left. CtAs, central arteries; hpf, hours post fertilization.

File name: Supplementary Movie 6

**Description: Brain capillary angiogenesis in control embryos (DMSO treated) - Zoom**

Zoom of the confocal time-lapse movie 5 showing the region displayed in Fig. 2e. As indicated for Movie 5, wild type embryo (DMSO treatment at 26 hpf) with GFP expression from *Tg(fli1a:lifeact-GFP)^mu240^* displays normal sprouting, invasion and anastomosis of two CtA sprouts. Confocal stacks were acquired in 15 min intervals. Dorsal view, with anterior to the left. CtAs, central arteries; hpf, hours post fertilization.

File name: Supplementary Movie 7

**Description: Brain capillary anastomosis is impaired after Wnt signaling inhibition by IWR-1**

Confocal time-lapse movie of a representative Wnt-depleted embryo (IWR-1 treatment at 29 hpf) with GFP expression from *Tg(fli1a:lifeact-GFP)^mu240^* starting at around 32 hpf shows normal CtA sprouting and sprout invasion, but impaired or largely delayed anastomosis of CtA sprouts. Confocal stacks were acquired in 15 min intervals. Dorsal view, with anterior to the left. CtAs, central arteries; hpf, hours post fertilization.

File name: Supplementary Movie 8

**Description: Brain capillary anastomosis is impaired after Wnt signaling inhibition by IWR-1 - Zoom**

Zoom of the confocal time-lapse movie 7 showing the region displayed in Fig. 2e. As indicated for Movie 7, Wnt-depleted embryos (IWR-1 treatment at 29 hpf) with GFP expression from *Tg(fli1a:lifeact-GFP)^mu240^* display normal sprouting, invasion, but impaired or largely delayed anastomosis of two CtA sprouts. Confocal stacks were acquired in 15 min intervals. Dorsal view, with anterior to the left. CtAs, central arteries; hpf, hours post fertilization.

File name: Supplementary Movie 9

**Description: Brain capillary angiogenesis in control embryos (mCherry^iEC^)**

Confocal time-lapse movie of a representative *Tg(fli1a:lifeact-GFP)^mu240^* embryo expressing mCherry in ECs after heat shock at 26 hpf (mCherry^iEC^), starting at around 34 hpf shows normal CtA sprouting, invasion and anastomosis of two CtA sprouts. Lumen formation occurred in average in less than 2 hours after cell-cell contact formation. Confocal stacks were acquired in 20 min intervals. Still images and quantifications were displayed in Fig. 2f. Dorsal view, with anterior to the left. CtAs, central arteries; hpf, hours post fertilization.

File name: Supplementary Movie 10

**Description: Brain capillary anastomosis is impaired after Wnt signaling inhibition by mCherry-dnTcf^iEC^ expression**

Confocal time-lapse movie of a representative *Tg(fli1a:lifeact-GFP)^mu240^* embryo expressing mCherry-dnTcf in ECs after heat shock at 26 hpf (mCherry-dnTcf^iEC^), starting at around 34 hpf shows normal CtA sprouting and sprout invasion, but delayed anastomosis of two CtA sprouts. Confocal stacks were acquired in 20 min intervals. Still images and quantifications were displayed in Fig. 2f. Dorsal view, with anterior to the left. CtAs, central arteries; hpf, hours post fertilization.

File name: Supplementary Movie 11

**Description: Normal brain capillary angiogenesis in heterozygous *ve-cadherin^ubs8^* mutant embryo**

Confocal time-lapse movie of a representative heterozygous *ve-cadherin^ubs8/+^* mutant embryo with GFP expression from *Tg(fli1a:lifeact-GFP)^mu240^* starting at around 32 hpf shows normal sprouting, invasion and anastomosis of CtA sprouts. Lumen formation occurred in a time-frame of wild type controls (see Movie 3, Fig. 2) Confocal stacks were acquired in 20 min intervals. Dorsal view, with anterior to the left. CtAs, central arteries; hpf, hours post fertilization.

File name: Supplementary Movie 12

**Description: Impaired brain capillary anastomosis in homozygous *ve-cadherin^ubs8^* mutants**

Confocal time-lapse movie a representative *ve-cadherin^ubs8/ubs8^* mutant embryo with GFP expression from *Tg(fli1a:lifeact-GFP)^mu240^* starting at around 32 hpf shows normal sprouting and invasion of CtA sprouts, but completely impaired anastomosis and lumen formation. Confocal stacks were acquired in 20 min intervals. Dorsal view, with anterior to the left. CtAs, central arteries; hpf, hours post fertilization.
